# Hybrid reasoning for perception, explanation, and autonomous action in manufacturing

**DOI:** 10.1038/s41467-026-72378-9

**Published:** 2026-05-18

**Authors:** Christos Margadji, Sebastian William Pattinson

**Affiliations:** Department of Engineering, University of Cambridge, Cambridge, UK

**Keywords:** Industry, Mechanical engineering

## Abstract

Industrial processes must operate robustly in unpredictable environments, where errors are costly and difficult to detect. AI-based control systems offer a path forward but typically rely on large, labeled datasets, limiting their generalization to variable, data-scarce settings. Foundation models promise broader reasoning and knowledge integration yet struggle to deliver the quantitative precision required in engineering. Here, we introduce Control and Interpretation of Production via Hybrid Expertise and Reasoning (CIPHER): a systems-level vision-language-action (VLA) framework designed for industrial perception, explanation and control. CIPHER integrates a process expert for quantitative characterization of system states with retrieval-augmented reasoning grounded in process physics and knowledge. This hybrid design enables strong generalization to out-of-distribution tasks, allowing the agent to interpret textual or visual inputs, explain its decisions, and autonomously generate precise machine instructions without explicit supervision. In this work, CIPHER is deployed within multiple manufacturing systems, demonstrating precise, context-aware, and transparent control, with potential for deployment in real industrial environments.

## Abstract (web summary)

Authors introduce a hybrid vision, language, and action system for manufacturing, integrating a CNN based process expert for precise perception and retrieval-augmented reasoning for contextual grounding. It demonstrates autonomous, interpretable control across various manufacturing tasks without large datasets.

## Introduction

Embodied agents perceive and act through a physical or virtual body, enabling interaction with and learning from their environment through sequential and synchronous sensory processing and decision-making. They typically integrate capabilities like language processing^1,2^, visual perception^3^, and action execution^4^, enabling decisions that are easily set and interpretable. Powered by their large foundational models, these embodied agents exhibit emergent capabilities, such as creative problem-solving and adaptability^5^, owing to training on diverse, web-scale data^6,7^. Such agents can autonomously explore and interact with several environments, iteratively improving their own capabilities through self-verification, in turn out-performing gradient-based optimization reinforcement learning strategies^8,9^.

Embodiment of intelligence in open-ended environments has demonstrated promise, particularly in virtual (e.g., navigating web browsers and game worlds like Minecraft) but also physical settings^9–11^. In developing embodied agents for applications like autonomous driving, there has been significant progress, owning to the extensive availability of computational resources and training data, justified by the large-scale and homogeneous nature of the problem^12^. Progress has also been made in developing general physical intelligence, which can handle multiple tasks (particularly pick-and-place or other forms of object manipulation) with little to no retraining, even for unseen hardware configurations^11,13^. Still, commercial deployment of such intelligent systems remains limited, primarily due to safety concerns (issues concerning alignment, accountability, liability) and other technical or operational barriers (such as the need for extensive computational resources). Some of these challenges echo Moravec’s paradox, the observation highlighting that tasks easy for humans can be disproportionately difficult for robots and vice versa^12,14^.

Research on deep learning (DL) for process monitoring and quality assurance in manufacturing has grown rapidly, though comparatively less attention has focused on quality and process control. In polymer additive manufacturing (AM), diverse sensors—including acoustic, inertial, pressure, and vision—have been used extensively for process monitoring and defect detection^15–20^. Related studies have further explored real-time defect correction and iterative process refinement for novel geometries and materials, particularly relevant for scalable production^21–23^. In metal AM, e.g., selective laser melting, DL has been used to analyze synchrotron X-ray and thermal imaging data for real-time detection of pore formation^24^. Feature fusion strategies have been explored as a way to integrate heterogeneous sensor modalities for improved state signatures^25^. In thermo-forming processes, DL has been used for frameworks which can suggest optimal process parameter changes based on a small number of images of the manufactured part^26^. High-precision subtractive manufacturing processes, like CNC machining, have benefited from vision-based thermal error modeling using transformers and deep anomaly detection approaches relying on spindle current signals^27,28^. In welding, the use of neuromorphic (e.g., event-based) vision sensors has shown promise for intelligent in-situ monitoring^29^.

Among these works there is a common limitation. Models are overspecialized, excelling only within previously encountered defect modalities, and lacking the causal reasoning and generalization capacity required to address novel scenarios. They also rely on large supervised datasets (often featuring millions of samples) that are impractical or impossible to acquire. Typically, these approaches also rely on quality metrics that are inherently quantitative in nature (e.g., precise process parameter prediction,) which typically reside in continuous spaces. Conversely, the state of the art in embodied AI, driven by large language models (LLMs) and, by extension, vision-language models (VLMs), does not generally excel in such domains^30,31^.

**Fig. 1:**
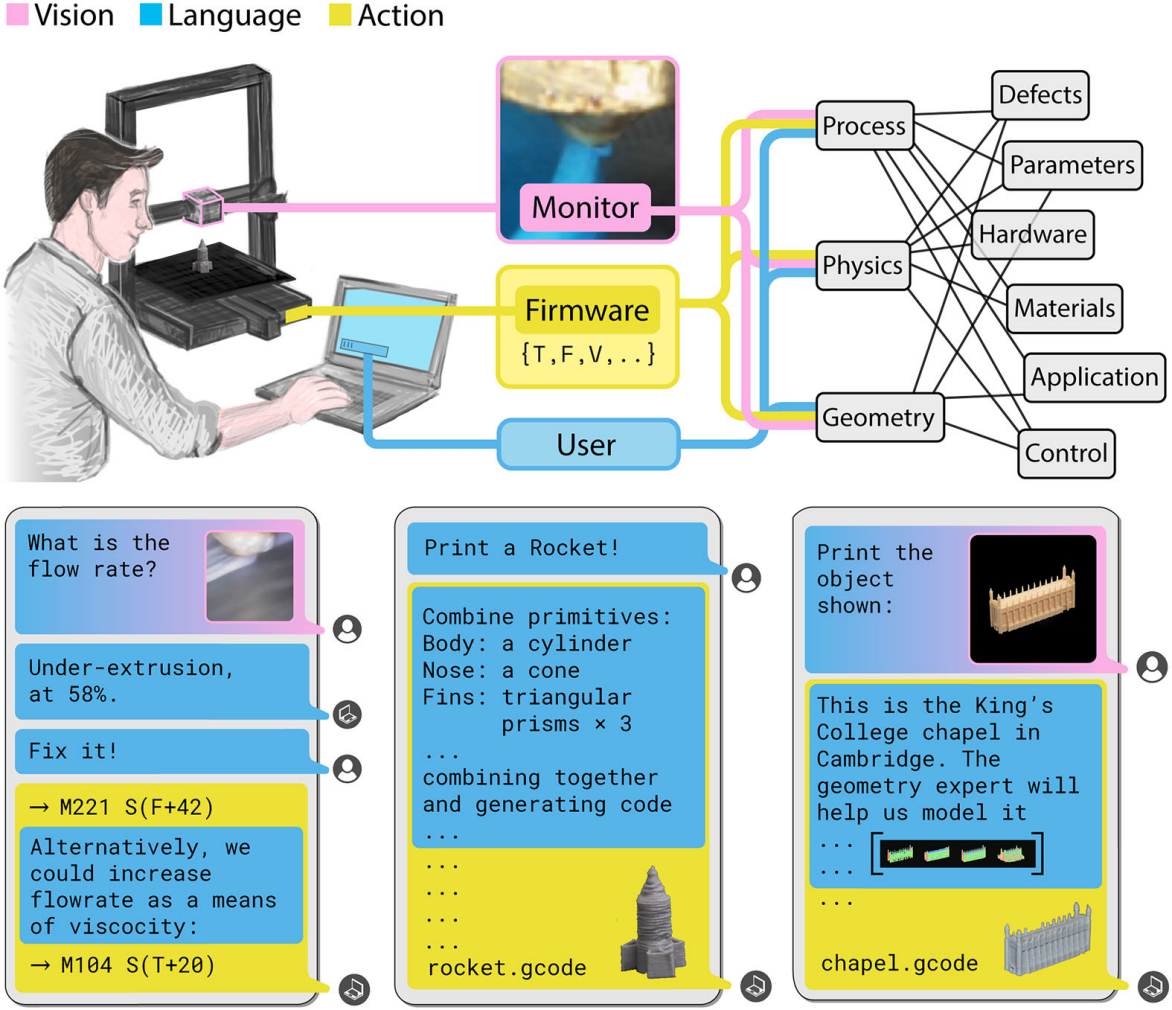
Overview of the CIPHER framework. CIPHER represents the orchestration of three experts: process, physics, and geometry, reflecting the primary lenses behind the science of manufacturing. In tandem, they enable precise perception with explainable, adaptable control through reasoning before action.

Here we report Control and Interpretation in Production via Hybrid Expertise and Reasoning (CIPHER), a vision-language-action (VLA) framework built upon the hybrid reasoning paradigm^32,33^. Rather than relying on large stochastic models for everything, CIPHER strategically employs compact networks for high-precision perception, while higher-capacity ones (LLMs) are reserved for reasoning and planning. Guided by the realities of industrial manufacturing, where data is scarce, processes complex, and reliability paramount, CIPHER is designed to be readily trainable and fast in inference, without compromising creativity or generalization to new errors and processes. At its core lies a process expert—i.e., a convolutional neural network (CNN) regressor—that augments perception by extracting task-specific quantitative features from visual signals, enabling accurate characterization of system states. This architecture combines the computational efficiency of traditional neural networks with the contextual flexibility of transformer-based large language models, achieving precise, engineering-grade perception alongside adaptive understanding and control. By integrating retrieval-augmented generation (RAG) with physics-informed chain-of-thought reasoning, CIPHER demonstrates emergent capabilities that allow robust adaptation to previously unseen control scenarios. While our primary experiments focus on fused filament fabrication (FFF) additive manufacturing, we further validate this component’s generality across other modalities, including machining, vacuum forming, and welding. Beyond its technical performance, analysis of CIPHER’s articulated reasoning enhances interpretability and may support operators in high-stakes industrial decision-making. Notably, CIPHER generalizes far beyond its training distribution; it can perform end-to-end autonomous fabrication directly from natural language or image prompts. It thereby emerges as a system capable of translating abstract human intent into concrete machine actions and, ultimately, tangible matter, without requiring any supervision. An overview of the CIPHER framework and its underlying functions can be seen in Fig. 1.

## Results

There is an evident discrepancy when publicly available multi-modal foundation models (GPT-4o, Llama-3.2) are tasked with reasoning across general and granular-level manufacturing tasks. While these models can effectively address queries pertaining to broad manufacturing contexts, they exhibit clear limitations when confronted with questions requiring macroscopic observations (Supplementary Fig. S1). This discrepancy stems primarily from the absence of high-quality manufacturing and process-monitoring data in the original training corpora: although such models may have encountered thousands of images of factory environments and their workers within, they have rarely been exposed to engineering-grade in-situ monitoring data. This lack of exposure constrains their ability to model the underlying physical and quantitative relationships that govern industrial systems, underscoring a key limitation to the deployment of current vision-language models in precision engineering.

The objective of this work is to develop a domain-grounded agent for intelligent manufacturing, embodied in CIPHER. The system is built on three guiding principles. First, CIPHER explicitly models contextual causality, recognizing that reasoning about physical processes requires more than visual pattern recognition. Second, its design reflects the realities of industrial data governance and privacy, where access to large volumes of high-fidelity process data is often limited; accordingly, CIPHER leverages background knowledge efficiently to reduce dependence on extensive task-specific supervision. Third, given the scarcity of machine-learning expertise in production environments, CIPHER prioritizes interpretable, knowledge-rich reasoning over complex or computationally intensive optimization. Together, these principles enable CIPHER to exhibit emergent capabilities, regulating previously unseen process parameters across diverse manufacturing settings and generating actuation commands for the machine’s kinematic systems. An image of the experimental setup and schematic of the desired workflow are shown in Supplementary Fig. S3a.

### Bringing engineering-grade perception in vision-language-action models

We detail our dataset sources in Methods. Given the absence of linguistic annotations within the original dataset (labels are continuous values, e.g. 30–300% flowrate), and the impracticality of manual annotation at scale, we systematically transform the collected numerical labels into structured natural language descriptions using predefined templates. This is followed by paraphrastic augmentation to increase linguistic diversity, as described in Methods and illustrated in Supplementary Fig. S2. Every generated caption contains a general statement about the process, followed by two statements that place the numeric parameter in meaningful context.

**Fig. 2:**
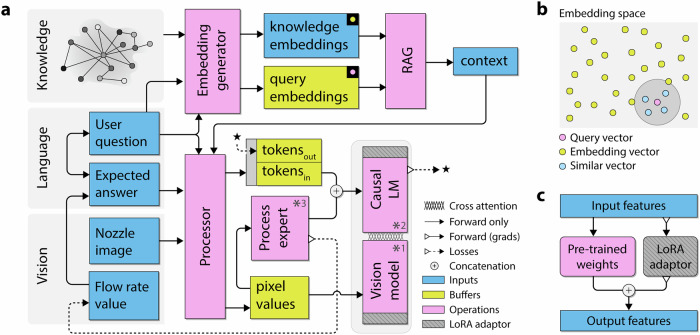
The agent architecture. **a** The VLM architecture integrates a process expert which enables good qualitative and quantitative alignment. Details of the architecture can be found in “Methods”. **b** Illustration of the embedding space generated during retrieval-augmented generation. The query vector (orange) represents the input, while embedding vectors (green) populate the space. Similar vectors (blue) are identified within a defined proximity (purple region) to the query, enabling the retrieval of relevant information. **c** Schematic representation of the low-rank adaptation filters. The adaptor is introduced in parallel to the pre-trained weights of the architecture, which remain frozen, while the adaptor itself is fine-tuned.

With these image-caption pairs, the objective centers on the capability to accurately reconstruct the synthesized sentences (or semantically equivalent alternatives) solely from visual input, thereby and most importantly, to retrieve the continuous values that have been embedded within. We formalize this perception problem in Supplementary Fig. S3. To this end, we train a model comprising a large language model (LLM) and a vision transformer (ViT), integrated via cross-attention layers as per the multi-modal Llama-3.2 framework, from which we also adopt pretrained weights^2^. Within this architecture, we also embed an additional convolutional neural network with residual connections between its blocks, specifically a ResNet-152 model (116m parameters) tasked to compress visual inputs into task-relevant features^34^. During inference, these features are linearly projected into token space and injected as a dedicated start token. Figure 2a summarizes the information flow between the architecture’s various components.

**Fig. 3:**
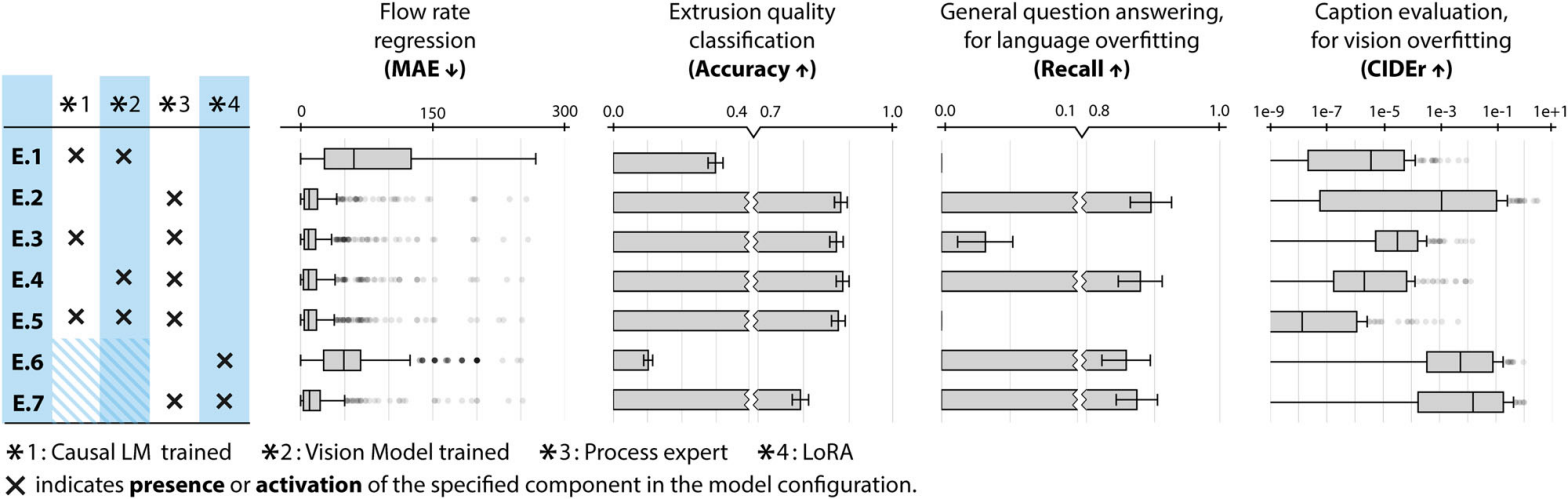
Results from ablation studies. We conduct experiments on variants of our architecture to evaluate qualitative and quantitative alignment, as well as the model’s susceptibility to catastrophic forgetting in both vision and language tasks. (error bars: standard deviation, 1000 samples).

In these experiments, our evaluation focuses on the model’s perception, in terms of quantitative and qualitative alignment. For qualitative alignment, we use classification accuracy to compare predicted classes against target classes mentioned in the generated sentences (e.g. image correctly associated with over-extrusion or not). For quantitative alignment, we extract from the text the continuous predictions and measure their discrepancy with the ground truth values using mean absolute error (MAE). We also assess vision and language overfitting as an indication of catastrophic forgetting. All reported metrics are calculated from 1000 held out test samples, sampled using a random split. The deployed evaluation metrics are formalized in Methods, results from the various experiments under different configurations are shown in Fig. 3, and a supplementary analyses and breakdown of the experiments are provided in Supplementary Table S1.

In the naïve fine-tuning experiment E.1 (without expert), achieving language format and general domain alignment is trivial. However, quantitative and qualitative information is not learned. Even when the entirety of the network is trained for an indefinite number of iterations, neither MAE nor classification accuracy decrease. This limitation is attributed to the classification-based loss function employed during gradient-based optimization, which is fundamentally based on discrete token spaces that lack semantic meaning, whose latent geometry is not optimized for numeric continuity. The non-deterministic nature of next-token prediction in the generation pipeline exacerbates the issue, though the problem persists even when the temperature is set to the limit of zero. This behavior is consistent with known limitations of large language models when it comes to regression. We elaborate on this in “Methods”.

**Fig. 4:**
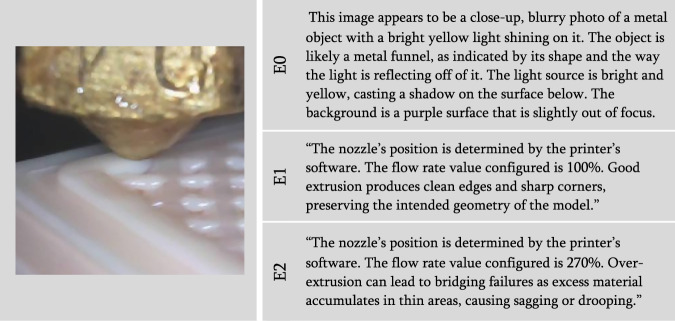
Captions generated for the shown image from three models. pre-trained (E.0), fine-tuned without (E.1) and with (E.2) process expert. The ground truth flow rate value is 300%.

On the contrary, in experiments where the process expert is activated, we report up to a five-fold reduction in MAE, compare results from E.1 (without expert) and E.5 (with expert) at 82.92 ± 71.83 and 17.62 ± 30.04, respectively. This is persistent across all tested settings, even when the vision and language modules are frozen, and only a small projection layer is optimized, that is, in experiment E.2. Sample answers from the pre-trained model, the trained model without, and the trained model with the process expert (E.0 to E.2) are shown in Fig. 4.

### Parameter-efficient fine-tuning

We further investigate parameter-efficient fine-tuning strategies by employing Low-Rank Adaptation (LoRA^35^, see “Methods”) in E.6 and E.7. By reducing the number of trainable parameters to only 2.4% of the model’s total, LoRA enables a 52.4% reduction in memory consumption (from 199.3 GB to 94.9 GB) and an 81% reduction in the amount of training data required to achieve comparable convergence. This efficiency aligns closely with factory conditions, where both computational resources and data availability are limited.

The distinction between models with and without a process expert remains evident in our LoRA experiments. Though we note that while LoRA demonstrates strong compatibility with the process expert, performance deteriorates by 7% and 9% on quantitative and qualitative metrics, respectively, when compared to the other strongest baselines. This degradation is plausibly a consequence of LoRA’s restricted parameter space, which limits network plasticity, an effect we later show can help mitigate overfitting and support generalization.

### Avoiding hard-coded perception at the cost of higher perplexity

**Fig. 5:**
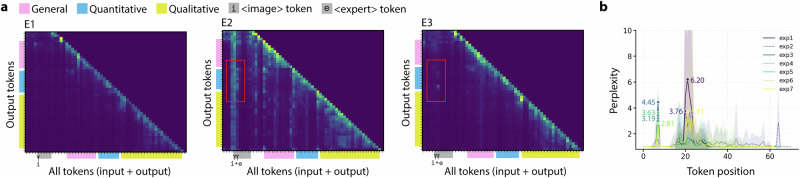
Perception analysis. **a** Sample attention rollout maps from E1, E2, and E3. **b** Average perplexity per token position (error bars: standard deviation, 100 samples).

Uncertainty in manufacturing must be carefully managed to avoid misaligned behavior. Using attention rollout (see “Methods”), we analyze how each preceding token contributes to the selection of the next token in the autoregressive setting, with sample attention maps in Fig. 5a (different normalizations in Supplementary Fig. S3b). We draw two key observations. First, models trained without a process expert (E.1) not only fail to predict accurate quantitative descriptors but also lose their ability to meaningfully attend to the image token. Interestingly, this does not hold true when LoRA is applied (E.6), perhaps because the limited parameter updates preserve perceptual grounding. Second, fine-tuning the causal language model shifts attention behavior: fine-tuned variants focus on the expert token only during quantitative prediction and never thereafter, in contrast, non fine-tuned models continue to reference it throughout inference. This indicates a more persistent integration of expert knowledge which raises confidence.

Next, we analyze token-level probability distributions and perplexity (Fig. 5b). In E.1, the model exhibits the greatest uncertainty, often defaulting to predictions near the mean of the target spectrum. Predictions have high mean surprisal (1.91 nats) and poor calibration (Brier score = 0.61), indicating both low confidence and weak discriminative ability. When the language model is fine-tuned (E.3), perplexity decreases markedly, with mean surprisal dropping to 0.95 nats and maximum token probability rising to 0.62. The resulting sharper probability distribution-driven by stronger attention on the expert token-renders temperature scaling (*T* < 1) largely ineffective. In contrast, when the LLM is not fine-tuned (E.2), perplexity remains higher (mean surprisal = 1.67 nats) but still below that of E.1. The model attends to the process expert token and distributes probability across a few plausible quantitative values, making its predictions more temperature-sensitive. Top-5 accuracy shows that even in this case, two or three candidate tokens account for over 99% of the probability mass, and these correspond to closely spaced values (e.g., 40.0 vs. 41.0), indicating that the residual uncertainty and higher losses stem from fine-grained numerical ambiguity rather than conceptual error.

### Prior knowledge is preserved

The preservation of reliable reasoning in data-sparse settings depends on retaining prior knowledge beyond what is seen during fine-tuning—a property of particular importance in manufacturing, where rich domain expertise coexists with limited task-specific data. To evaluate the extent of catastrophic forgetting, we compared the pre- and post-fine-tuning performance of the models on standard natural language processing benchmarks, using 100 randomly sampled items from the SQuAD question-answering dataset and the Flickr30k image-captioning dataset^36,37^. Recall and CIDEr were selected as validation metrics (see Methods).

Baseline performance (E.0) yielded values of 0.965  ± 0.015 for Recall and 0.412  ± 0.433 for CIDEr. In experimental configurations E.1 and E.5, we observed pronounced catastrophic forgetting. This manifested as an almost complete degradation of the evaluated scores: when presented with out-of-distribution inputs such as images from Flickr30k, the models generated captions overly similar to those seen during fine-tuning, suggesting severe overfitting. When only a single modality was fine-tuned (and in the absence of LoRA), catastrophic forgetting persisted but was notably less severe, affecting the tuned modality more strongly (i.e., vision performance degraded when the vision component was adapted, and vice versa for language). The introduction of LoRA in E.7 substantially mitigated these effects, preserving 93.2% and 95.6% of the baseline Recall and CIDEr values.

Among all configurations, E.2 proved the most robust to catastrophic forgetting. In this setup, only the terminal projection layer of the vision module was adapted, while the core vision feature extractor and language model parameters remained intact. This selective adaptation not only minimizes computational overhead but also appears to preserve the model’s representational balance. By mitigating catastrophic forgetting, E.2 retains more natural and contextually grounded attention patterns, avoiding the rigid, hard-coded perception observed in more aggressively fine-tuned models. This property is particularly advantageous in manufacturing contexts, where systems must continuously integrate prior domain knowledge while adapting to new tasks, materials, processes without retraining from scratch. Consequently, E.2 was selected as the preferred configuration for subsequent analyses and might be referred to as ’Ours’ hereafter.

### Embodying CIPHER for action generation in novel settings

**Fig. 6:**
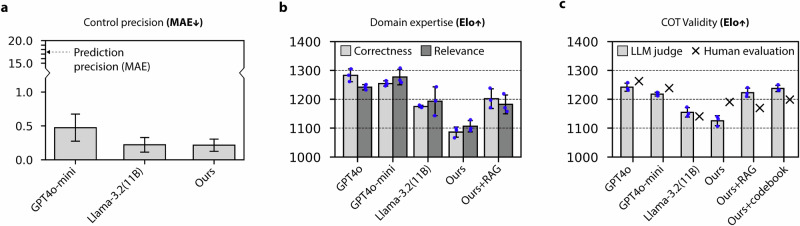
Control performance of various agents across different scenarios. **a** MAE for in-distribution tasks (error bars: standard deviation, 1000 samples). **b** Elo ratings for QA tasks (correctness, relevance; error bars: standard deviation, 3 rounds, 100 questions each). **c** Elo ratings for final control command quality in out-of-distribution control scenarios (COT validity, error bars: standard deviation, 3 rounds, 100 challenges each).

We start with traditional control. CIPHER in our experiments demonstrated high accuracy in predicting flow-rate values and articulating them well in natural-language format. It also retained its pretrained language-generation capabilities, showing only minor degradation following fine-tuning. To extend this evaluation, we examined CIPHER’s ability to generate executable machine-code instructions, recognizing that control languages such as G-code (ISO 6983) constitute a natural extension of linguistic abstraction. In this experiment, Prompt S1 (supplementary files) instructed the model to perform a numerical computation and produce a structured M221 SN command, where N specifies the required flow-rate adjustment. This task evaluated two essential competencies: linguistic reasoning and symbolic computation, both critical in industrial contexts. Across 100 synthetic test cases, CIPHER achieved performance on par with LLaMA 3.2 and slightly exceeded GPT-4o-mini in accuracy (Fig. 6a), although all models demonstrated generally adequate performance for the task. The resulting actions introduced only a negligible deviation from target flow-rate values ( ≈ 1.2% degradation relative to baseline). Latency analysis showed end-to-end correction frequencies around 2.3 ± 1.2 Hz, comparable to traditional controllers. While classical systems may still respond faster, CIPHER provides the added benefit of interpretable, language-based reasoning, bridging symbolic understanding before arriving to direct physical control.

We hypothesise that domain contextualisation—implemented through the injection of problem-specific and process-relevant manufacturing and engineering knowledge—augments CIPHER’s internal process understanding and leads to more robust emerging behaviours. In particular, the form and value of this contextualisation will depend on both the problem being solved and the specific process being operated by CIPHER, especially in settings where direct one-to-one knowledge is scarce and exhaustive scenario testing is infeasible.

To systematically assess this on domain-specific knowledge, we curated a set of 100 textbook-style questions derived from relevant manufacturing and materials science literature. These questions were posed to CIPHER and a set of established baselines, with comparative outcomes summarized in Fig. 6. To enhance contextual grounding, we integrated a RAG module (schematics in Fig. 2b; see “Methods”) which dynamically retrieves semantically aligned content from a structured knowledge repository. This repository was constructed by traversing the hierarchical mind-map presented in Supplementary Fig. S4, populated through LLM-assisted generation of factual statements. All contents of the knowledge repository were validated through human experts to ensure correct alignment and comprehensive topic coverage.

For a statistically robust comparison, model responses were evaluated using the Elo rating system—a framework originally developed for competitive ranking (e.g., chess)^38^ and now widely adopted for pairwise LLM benchmarking. The evaluation protocol is summarized in Methods, with results averaged across three independent rounds (Fig. 6b; Supplementary Table S2). Although minor evidence of catastrophic forgetting was observed in the modified model (Ours), integration of the RAG module (Ours+RAG) effectively mitigated this effect. More importantly, contextual grounding through RAG produced a net improvement in factual accuracy, enabling the model to surpass its original performance baseline and outperform LLaMA 3.2 in overall correctness.

We observe that the emergence of novel control capabilities is a function of process understanding. CIPHER’s capacity for generating corrective strategies in novel scenarios was tested using prompts that demanded more comprehensive planning beyond basic flow-rate adjustments (see Methods). To compare our agent with other candidates, we independently prompted all to analyze randomized firmware states of the manufacturing system (see Methods) and produce structured outputs comprising: (a) a chain-of-thought reasoning paragraph, followed by (b) a proposed control command. The former promotes causal analysis of faults, while the latter converts these insights into targeted system actions; broadly, how experienced factory operators may diagnose and resolve process malfunctions through precise corrective commands.

We first validated the quality of the models’ outputs using the Elo ranking system, with results summarized in Fig. 6c and detailed in Supplementary Table S2. Rather than comparing intermediate reasoning chains, we opted for final-answer evaluation to mitigate potential bias arising from differences in linguistic style, fluency, and structure, and rather focus on technical correctness. Consistent with prior findings, our RAG model outperforms Llama, and the overall trend revealed a positive correlation between robust question-answering ability and enhanced physics-informed reasoning. This supports our hypothesis that domain-specific knowledge injection reduces hallucinations and strengthens problem-solving in unfamiliar contexts. We also show that providing CIPHER with a detailed G-code playbook (i.e., more relevant context, see Methods) further improved its performance (Elo_Gcode_ = 1238 ± 9), elevating the agent above GPT-4o-mini (Elo_GPTmini_ = 1218 ± 5) and achieving near-parity with GPT-4o (Elo_GPT4o_ = 1241 ± 11). A human-evaluation study (see Methods) involving 10 subjects corroborated these findings, showing strong agreement between human expert judgment and LLM-based pair-wise ranking.

On a qualitative note, we have seen occasions where CIPHER came up with valid multi-step strategic control plans. In instances, it adjusted nozzle temperature to modulate flowrate, by autonomously leveraging the temperature-viscosity relationship (reasoning: “[…] *Increase temperature to enhance melting efficiency and reduce flow resistance* […]”.) It accurately identified mismatches between prescribed nozzle temperatures and material-specific melting points, such as recommending 240 ^∘^C for ABS when it was 220 ^∘^C, and suggested corrective adjustments with the M104 (Set Hotend Temperature) command. When processing flexible filaments like TPU, the agent proactively reduced feed rate to prevent filament buckling. At times, CIPHER proposed adjusting fan speed to reduce stringing artifacts and prudently withheld action when no correction was needed. In one remarkable case, it advised aborting the print after determining that the current parameters had likely degraded the process beyond recovery.

To assess the generalization of CIPHER’s control capabilities beyond AM, we extended our evaluation to previously unseen processes, including machining, vacuum forming, and welding (see “Methods” and results in Supplementary Fig. S6). In each case, the agent was tasked with diagnosing process anomalies and proposing corrective actions without any retraining or process-specific supervision. Despite the absence of prior exposure to these domains, CIPHER leveraged its preserved background knowledge and strongly attended to the provided context to generate physics-consistent reasoning and viable control strategies. Across all tasks, the RAG-augmented configuration consistently outperformed the our base model, achieving performance nearly on par with GPT-4o. These findings suggest that contextual knowledge retrieval, when combined with structured manufacturing knowledge, can enhance the adaptability and reasoning performance of intelligent agents across diverse industrial processes.

### Printing without explicit geometric supervision

**Fig. 7:**
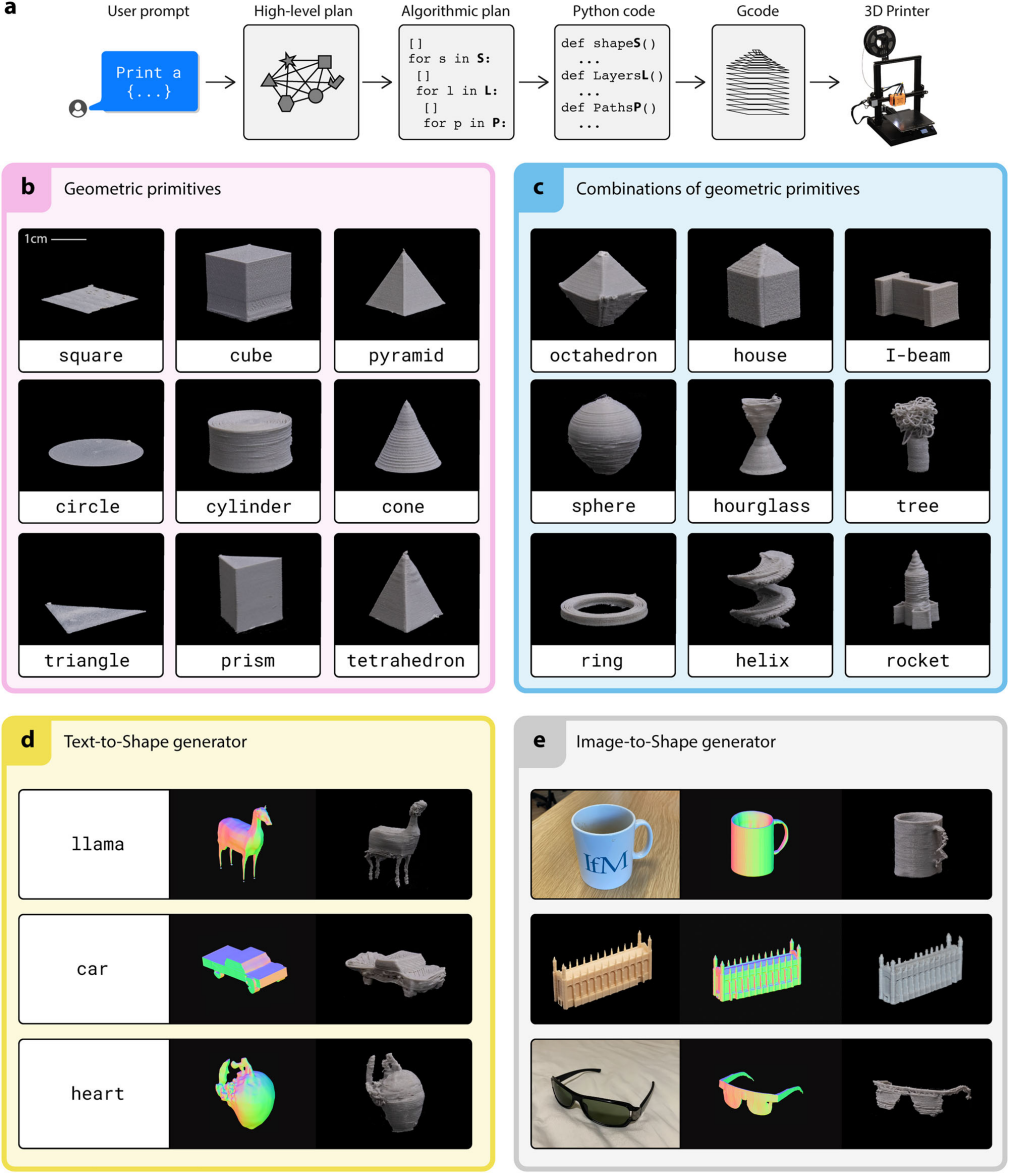
Overview of our geometry experiments. **a** The workflow for geometric requests. **b** Simple shapes are handled by simple, well-defined geometric primitives. **c** Requests of medium complexity are constructed as combinations of geometric primitives. **d**, **e** Complex requests are handled by the 3D shape generator.

We finally extend our evaluation to test CIPHER’s capacity to interpret and manipulate machine code that governs not only process parameters but also the kinematics of the manufacturing system itself. The aim is to demonstrate how foundational knowledge can reduce dependence on human expertise, extending the agent’s role from parameter-level quality control to broader process control. We hypothesize that CIPHER can decompose complex geometries into assemblies of simpler primitives, enabling the generation of manufacturable designs directly from abstract geometric concepts. This capability is supported by an incrementally expanding repository of validated functions, which grows only when preceding tasks are successfully completed, allowing the agent to accumulate verified operational knowledge over time. We achieve this using prompts that lead into chain-of-thought reasoning patterns resembling the workflow depicted in Fig. 7a and further detailed in Methods. This workflow structure was carefully crafted to closely parallel contemporary manufacturing pipelines.

Simple requests were addressed in a one-shot manner. A key factor contributing to this success was the initial focus on simpler single-layer primitives (e.g., 2 × 2 cm square), which formed the basis of multi-layer ones, once they were added in the skills library. The inclusion of this library proved highly beneficial, as it provided computational shortcuts, enabled iterative learning, and standardized coding style. For instance, a 2 × 2 × 2 cm cube was inferred as a stack of 100 × 2 × 2 square layers (assuming a nominal layer height of 0.2 mm), a relationship that became straightforward once the corresponding function for square generation was readily available. Similarly, a cone was synthesized by linearly stacking progressively smaller circles.

Synthesizing and printing more complex 3D assemblies by combining multiple primitives proved both challenging and feasible. CIPHER was able to reason compositionally, describing objects as assemblies of simpler elements, e.g., a rocket as an assembly of tetrahedral fins, a cylindrical body, and a conical tip. However, it often struggled with precise spatial alignment and orientation of the individual pieces. Interestingly, more complex structures such as the helix (Fig. 7c) were generated flawlessly, suggesting that explicit mathematical regularity enables more stable spatial reasoning than loosely defined alignments. With more guidance, CIPHER was able to progressively arrive at increasingly complex structures, including fully threaded bolts and complex mechanical gears, Supplementary Fig. S7b. In cases, the agent failed to add any support structures (see Sphere), although it was capable of reorienting parts to minimize overhangs (see I-Beam). In some cases, the agent demonstrated solutions that exceed human creativity, such as generating randomized extrusion paths to emulate nature’s irregularity (e.g., see leaves on the tree).

Repeatability experiments revealed that CIPHER’s responses were not strictly deterministic; when presented with identical prompts, the agent occasionally produced distinct yet equally valid geometric solutions. This variability reflects a form of controlled creativity rather than inconsistency, suggesting that sampling parameters could be tuned to balance determinism, which is essential in engineering contexts, against creative exploration, which may prove valuable during design ideation. Notably, the model’s outputs were expressed as highly parameterized Python functions, providing explicit control over geometric dimensions unattainable by current generative shape models. We also demonstrate that infill type itself can be treated as a design variable, with functionally graded properties-such as controlled extrusion gradients-readily attainable (Supplementary Fig. S7a). Such parameterization is highly valued in engineering practice, as it enables systematic and exhaustive exploration of the design space, particularly within simulation environments.

In theory, any shape can be constructed by combining simpler primitives, but this might become computationally intensive and impractical for complex designs. In our experiments, assemblies involving more than four types were observably harder to execute (see Duck, Supplementary Fig. S7c). As a fallback to address this, we integrated in the system an implicit shape generator which can be conditionally called, see Methods. This fallback enabled shapes that are difficult to express through primitive composition alone, including cavity-like internal structures in the human heart example. However, its outputs were often only visually plausible: because the model lacked an explicit representation of fabrication constraints, it frequently produced mechanically fragile geometries that violated basic process requirements, such as minimum feature size (for example, the legs of the llama) and support constraints for overhangs (for example, the temples of the sunglasses). By contrast, in cases where the fallback was inadequate, the block-based approach more reliably satisfied these constraints (see table in Supplementary Fig. S7c).

## Discussion

This work introduces CIPHER, a vision-language-action (VLA) system designed to support autonomous control in data-limited, high-precision manufacturing environments. While large foundation models have achieved remarkable generalization in language and vision, their application to physical domains remains constrained by limited capacity to represent and reason over continuous, domain-specific parameters. CIPHER addresses this gap through a modular framework that integrates vision-based perception, natural language reasoning, and control, bridging the divide between abstract intelligence and physical execution. The system performs both perception and decision-making while maintaining interpretability and generalization, two properties often in tension in industrial AI.

Central to our contribution is a modular systems architecture that couples a large vision-language transformer with a specialized process expert and retrieval-augmented reasoning. This integration enables reasoning over continuous-valued physical states (such as material flow rate) that are critical in manufacturing yet poorly represented in existing architectures. The expert component enhances both quantitative prediction and semantic alignment between language and system state, demonstrating the effectiveness of combining pretrained models with targeted modules when precision is paramount. Importantly, this approach circumvents the need for large annotated datasets, aligning with the reality of most manufacturing contexts, where labeled data are limited and costly to acquire.

The retrieval-augmented module allows the system to respond coherently to novel control scenarios and novel processes without direct supervision, leveraging external domain knowledge to generate physics-informed chain-of-thought explanations and actionable plans. This is a marked departure from prior data-driven controllers, which tend to generalize poorly outside training distributions. CIPHER’s capacity to produce interpretable rationales supports more transparent decision-making, although we realise the reliability of these explanations in safety-critical scenarios warrants further scrutiny. Going forward, achieving manufacturing-grade reasoning will likely require explicit formal representations of process knowledge, causal models linking interventions to outcomes, tighter integration with manufacturing standards and controller semantics, temporal reasoning over machine histories, and calibrated uncertainty quantification for both state estimation and recommended actions.

CIPHER represents a systems-level contribution toward machine learning models capable of directly manipulating matter and yielding physical parts through abstract instructions. While it can decompose geometric prompts into manufacturable primitives and generate corresponding G-code that is directly executable, its spatial reasoning remains limited, particularly in accounting for implicit constraints such as support structures, minimum feature size, and print orientation. Failures in complex geometries indicate that integrating manufacturing-aware priors will be essential for improving robustness in autonomous fabrication. More broadly, our findings highlight the need for spatially aware language models and manufacturing-constrained 3D shape generators that can more effectively address this family of tasks, bridging creative geometric synthesis with the practical constraints of physical realization.

More broadly, this work raises questions about the role of modularity in embodied AI. CIPHER’s architecture suggests that targeted integration of process-expert and foundational components can offer a pragmatic alternative to end-to-end training when generalization and precision must coexist. At the same time, reliance on explicit module boundaries introduces interface challenges, for instance, ensuring semantic alignment between expert predictions and language model outputs, which merit closer attention in future research. Nonetheless, these results suggest that foundation models, when appropriately constrained and supported, can move beyond abstract reasoning to support physically grounded, precise decision-making in real manufacturing environments.

## Methods

### Dataset

#### Vision

We use the dataset from ref. ^23^. A commercial endoscope is mounted on the head of the printer, capturing nozzle images as material is being deposited. These visual data streams are synchronized with corresponding real-time process parameters recorded from the printer’s firmware, for instance, flow rate value, hereby treated as our labels. This leads to image–parameter pairs which allow for one-to-one mapping.

The original dataset comprises more than five million nozzle images, but during pre-processing, more than 80% of the samples are discarded due to being too blurry or overly like others, ensuring higher quality and diversity in the remaining data. After quality filtering, 920,000 image-parameter pairs were retained and split 80/10/10 for training, validation, and testing. All splits were performed with a fixed random seed to ensure reproducibility, and data are available as per the data availability statement below.

During data loading, photometric and geometric augmentations are applied on a per-batch basis to increase robustness to varying conditions (e.g., lightning conditions, different camera angles) These augmentations included colour jittering (0–5% brightness, contrast, and hue shifts), rotations (−20^∘^ to 20^∘^), translations (−10 to 10 pixels in both *x*- and *y*-axes), magnifications (80% to 120%) of the original size, and random horizontal flipping (with *p* = 0.5).

#### Language

##### Process perception

In the dataset’s original format, each image is paired with a flowrate value retrieved from the firmware of the 3D printing system at the time of capture. To frame the problem as a vision-language task, we convert the flowrate value (our quantitative descriptor) into a natural language caption. Each of the captions includes:

- A general statement about 3D printing.
- A quantitative sentence specifying the exact flowrate value.
- A qualitative descriptor based on the flowrate value: under-extrusion ( < 90%), good extrusion ( ≈ 100%), or over-extrusion ( > 110%).

For each caption component, a template is randomly selected from a pre-defined set to prevent overfitting by diversifying. This maintains linguistic variability while ensuring accurate representation of the quantitative data.

##### Knowledge for retrieval-augmented generation

We employ retrieval-augmented generation (RAG) for question answering to enhance physics-intuitive chain-of-thought reasoning. To integrate domain knowledge into the pipeline, we first construct a comprehensive mind map of the 3D printing domain. This mind map serves as a foundation for generating relevant facts. In total, 3930 facts are generated, covering a diverse range of topics—from the chemistry of the materials involved to more specialized subjects, such as the history of the technology. These are converted as embeddings using Ada-v2 provided from OpenAI’s API once and saved for future retrieval. The embedding space is plotted in 2D in Supplementary Fig. S5 using the t-SNE dimensionality reduction algorithm. We observe overlap between specific categories, such as physics and process parameters, offering intriguing insights into their interconnectedness.

During inference, when RAG is enabled, questions are encoded using the same embedding model, and cosine similarity identifies the N nearest facts (*N* = 5 unless otherwise specified). These facts are then incorporated into the system message as additional context and passed to the agent to answer the original question. In control scenarios where the question is too vague to yield a meaningful embedding, we apply RAG in a cascaded manner: the control problem is first evaluated without RAG, and the resulting reasoning is then used to retrieve relevant context before re-evaluating the control problem again, this time with the added context.

#### Action

To facilitate accurate G-code generation, we parse the open-source Marlin firmware documentation (https://marlinfw.org/meta/gcode/) into a JSON format, where each key corresponds to a command name and its value includes a brief description and a URL (e.g., https://marlinfw.org/docs/gcode/G000-G001.html). Similar to the RAG pipeline, during inference the agent reasons over a task, converts the reasoning into an embedding using Ada-v2, retrieves the most relevant command using cosine similarity, and incorporates the fetched command’s usage notes for generating precise G-code instructions.

### Agent architecture

Our agent comprises of 3 main experts, namely process expert, physics expert, and geometry expert. The process expert is responsible to perceive and understand the process, while the physics expert is responsible to turn observations into reasoning and actionable decisions. The geometry expert is responsible to handle geometric requests.

#### Physics expert

Our physics expert is fundamentally based on the Llama-3.2-11B-Vision ^2^ (https://huggingface.co/meta-llama/Llama-3.2-11B-Vision) architecture, with the pre-trained weights loaded and used from the available public release. The architecture comprises an image encoder, a projection layer, and a large language model (LLM). The image encoder, in our case a vision transformer ViT-H/14 trained with the contrastive objective^39^ (https://huggingface.co/laion/CLIP-ViT-H-14-laion2B-s32B-b79K), transforms inputs from pixel space into a high-dimensional feature space, while the projection layer maps these features into LLM-compatible tokens, enabling seamless concatenation with the actual language tokens for downstream processing and reasoning.

During training, the model is trained end-to-end using an autoregressive next-token generation strategy, optimized with a cross-entropy loss function. At inference time for vision tasks, a processor prepares the image and attempts to reconstruct the reference caption, as defined in Methods. At inference time for language tasks, the input prompt is encoded, and the answer is generated in an auto-regressive fashion. Each token in the vocabulary is assigned a probability based on the model’s predictions, and, following standard LLM practices, the next token is selected.

##### Parameter-efficient fine-tuning

We deploy low-rank adaptation (LoRA) for parameter efficient fine-tuning and hyperparameters are detailed in Supplementary Table S3. While 4-bit quantization was explored to reduce the model’s memory footprint, it significantly deteriorated the effectiveness of the process expert. This degradation is likely due to compatibility issues, as the vision components of the processor’s outputs serve as the inputs to the process expert. The reduced precision in 4-bit quantization introduces mismatches that disrupt this interaction, ultimately impacting the model’s ability to maintain accurate and reliable performance.

#### Process expert

The process expert is a residual neural network ResNet-152, with 116 million parameters (https://huggingface.co/microsoft/resnet-152). Its primary role is to take as input a nozzle image of dimensions 224 × 224 × 3 and explicitly predict the associated flow rate value. To adapt the architecture for this task, the final classification layer is replaced with a linear layer that maps 1024 features to a single output neuron. To fully leverage the pretrained weights, mean-std normalization is applied using the original statistics from the ImageNet dataset. Furthermore, the output space is transformed into logarithmic space, effectively mapping flow rate values from 30 to 300 to approximately −1 to 1.

During training, a batch of images is processed by the Llama processor and subsequently passed to the process expert. The expert generates predictions, and the ground truth values are used to perform an optimization step on the process expert. An AdamW optimizer is used, and a linear learning rate scheduler (*γ* = 0.5) is implemented with original value 10^−4^. The updated predictions are then re-evaluated and converted from floating-point values to tokens, which are returned as input to the LLM alongside the original vision and prompt tokens. This iterative process ensures tight integration between the process expert and the LLM, enabling improved downstream performance.

#### Geometry expert

Our central hypothesis posits that the reasoning module can decompose complex geometries into constituent assemblies of simpler primitives. We further contend that such primitives, typically expressed with rigorous mathematical or algorithmic representations, can be formally described through well-defined mathematical functions. CIPHER’s coding skills translate these mathematical function into executable Python code, which runs. We grant the agent access to a terminal environment in which the Python code can then be executed, and the output is the machine instructions in the form of a .gcode file. The file is subjected to verification before being transmitted to the additive manufacturing (AM) system for printing.

##### Complexity tests

All geometry requests undergo a complexity test to determine which branch of the geometry expert will handle the task. The complexity test leverages GPT-4o’s reasoning capabilities to assess whether a shape can be constructed using a combination of geometric primitives. Tasks requiring more than four geometric primitives are considered too complex and are routed to the text-to-shape generation module. Similarly, any request involving printing from images is sent directly to the image-to-shape generation branch. We note this is not an essential step of our pipeline but we introduce it as a fallback strategy, allowing us to draw some future directions regarding manufacturing-aware shape generators.

##### Shape building

For simple shapes, involving geometric primitives or their combinations, requests are reformatted based on Prompt S5 (supplementary files) and processed by the geometry module. As successful geometric operations are performed, the corresponding functions that generated these successful operations are added to the “useful functions" division of the prompt. This approach builds a growing library of reusable, validated functions, enabling more efficient handling of future tasks by reducing redundancy and ensuring consistency. The prompt, enhanced by the accumulated “useful functions," is submitted to the GPT-4o API, which generates Python code automatically formatted into a .py file. This file is executed to produce a G-code file tailored to the requested operation. Before being forwarded to the 3D printing system, the generated G-code undergoes a validation step using the Cura Engine to ensure its executability and compatibility with the 3D printer. Hardware actuation was performed under human supervision with automatic emergency stop interlocks.

##### Shape generation

For complex text-to-shape and image-to-shape requests, we use Shap-E (https://huggingface.co/openai/shap-e) with pipeline visualised in Supplementary Fig. S7e. Shap-E generates implicit neural representations of 3D shapes, representing objects as continuous fields that determine whether a point is inside, outside, or on the shape’s surface. These representations are rendered into discrete 3D meshes using the marching cubes algorithm. To enhance user control, the system samples four shape options for each request, see Supplementary Fig. S7d, allowing the user to review and select their preferred design. The chosen shape is then passed to the subsequent module for G-code generation and, finally, to the 3D printer for execution.

#### Training

During training, we feed the model a concatenated tensor (expert + vision + text tokens) and apply a causal attention mask so that each position only attends to itself and earlier positions. The training objective then becomes to minimise the negative log likelihood of the ground truth sequence, optimising for the cross entropy loss of all following positions. All components: the vision encoder, projection layer, the process expert, and the language backbone are trained fully end-to-end; more details for training and different configurations follow in Methods.

We train our agent end-to-end with a single node of 4  × NVIDIA A100-SXM-80GB GPUs, supported by 1 TB of memory. Fine-tuning the model takes 20 hours over a single epoch which has been shown adequate for convergence. Hyperparameters and important network configuration settings are detailed in Supplementary Table S3. Other details regarding training, inherently related to the selected architecture may be found in the original Llama paper and our codebase^2^.

### Experiments

#### Machine instruction generation

We evaluated three models (CIPHER (E.2 configuration), LLaMA 3.2, and GPT-4o-mini) on a machine-code generation task designed to assess symbolic reasoning and control-language proficiency. Each model was prompted identically using Prompt S1 (supplementary files), which instructed it to perform a numerical computation and output a structured command of the form M221 SN, where N specifies a flow-rate adjustment percentage according to the ISO 6983 G-code standard. A total of 100 target value estimates were uniformly sampled from the interval [30, 300], consistent with the empirical distribution of flow-rate values in the original dataset. To account for measurement uncertainty, corresponding firmware values were synthetically derived by perturbing each estimate within the empirical mean absolute error bounds of 17.52  ± 28.89 in E.2. The resulting 100 (estimates, firmware) pairs were used as in the defined placeholders within the prompt, and all models were instructed to converge on a target flow rate of 100%.

#### Novel control tests

We further evaluated CIPHER using a benchmark of 100 randomized control scenarios, each defined by unique combinations of system parameters, including nozzle temperature, feed rate, Z-offset, and working material, while maintaining a constant target flow rate of 100%. This setup explicitly incentivized reasoning about alternative root causes of process deviation rather than merely compensating through extrusion adjustments. Each case was provided as system in Prompt S2 (supplementary files) and evaluated across all candidate models under identical prompting conditions. This experiment was designed to test adaptive reasoning and corrective planning under novel process configurations.

#### Emerging control in novel processes

We first compiled optimal process parameter settings from textbooks, technical manuals, and online manufacturing forums, and subsequently verified them through interviews with respective domain experts. Specifically, we collected validated parameter ranges for machining (spindle speed, feed rate, depth of cut, coolant) across materials including copper, stainless steel, aluminum, and titanium; for vacuum forming (heating temperature, heating time, vacuum time) across ABS, PVC, PETG, acrylic (PMMA), and polypropylene; and for welding (current, current type, gas flow, gas type, back purge) across aluminum, carbon steel, stainless steel, titanium, and copper. To generate control scenarios that required corrective reasoning, we randomly selected one material per process and adversarially modified one of its optimal parameters. This procedure was repeated 100 times for each process, yielding 100 unique control scenarios per process. Knowledge bases for machining, vacuum forming, and welding were constructed following the same methodology used for additive manufacturing, combining curated literature, expert input, and structured fact search. The resulting parameter sets were inserted into the defined placeholders within the prompt.

### Validation and benchmark metrics

#### Mean absolute error

To evaluate the quantitative accuracy of predictions, we calculated the mean absolute error (MAE) between the predicted flowrate values and the ground truth values obtained from the 3D printing firmware. The MAE is computed as:

1 $${{{\rm{MAE}}}}=\frac{1}{n}{\sum }_{i=1}^{n}\left|\widehat{{y}_{i}}-{y}_{i}\right|$$

where *y*_*i* represents the ground truth value, $\widehat{{y}_{i}}$ represents the predicted value, and n is the total number of samples. This metric provides an intuitive measure of prediction accuracy, as lower MAE values correspond to better agreement between predicted and actual flowrates.

#### Language recall

To evaluate the alignment between predicted and ground truth language responses, we compute recall as the fraction of tokens in the ground truth that are correctly predicted by the model. Tokenization is applied to both predictions and references, and recall is calculated as:

2 $$R={\widehat{T}}_{correct}/| T|$$

where $\widehat{T}\_correct$ is the number of correctly predicted tokens and $\left|T\right|$ is the total number of tokens in the ground truth reference. If the ground truth contains multiple references, the highest recall among them is used. This metric emphasizes the model’s ability to capture essential elements of the reference text.

#### CIDEr score

Consensus-based image description evaluation (CIDEr) was developed to evaluate generated image captions by measuring their similarity to human-written reference captions. In the context of text-generation tasks (e.g., describing the quality of extrusion), CIDEr provides a score indicating how closely a generated response aligns with reference descriptions. Higher CIDEr scores indicate closer agreement with the reference text. It can be calculated using equation (3)

3 $$CIDE{r}_{n}({c}_{i},{S}_{i})=\frac{1}{m}{\sum }_{j}\frac{{g}^{n}({c}_{i})\cdot {g}^{n}({s}_{ij})}{| | {g}^{n}({c}_{i})| | \,| | {g}^{n}({s}_{ij})| | }$$

where *c*_*i*_ is the generated caption, *S*_*i*_ is the set of reference captions, *g*^*n*^(*c*_*i*_) is the n-gram frequency vector of *c*_*i*_ and ${g}^{n}\left({s}_{ij}\right)$ is the n-gram frequency vector of the *j*-th reference text. *m* is the total number of available reference captions (in our experiments, *m* = 5 as commonly used with the Flickr30k dataset) and ∥•∥ represents the norm of the vector, ensuring proper normalization.

#### Qualitative-quantitative alignment

The qualitative-quantitative alignment has been designed to evaluate whether the predicted flow rate aligns with the described extrusion quality in the generated text in a classification fashion. Specifically, it checks if a flow rate below, equal to, or above 100% is correctly described as under, good, or over-extrusion, respectively. Alignment can then be expressed as a ratio of correct predictions to the number of total predictions.

#### Attention rollout

Attention weights are often employed as explanation probes to reveal what information is propagated between layers in a transformer model. However, directly extracting these weights produces *N* distinct attention matrices, corresponding to the number of heads in the transformer architecture. To obtain a more holistic view of the model’s internal information flow, we apply a post-hoc method known as attention rollout^40^. This method aggregates the multiple attention matrices across layers and heads, thereby quantifying the flow of information through self-attention mechanisms. The resulting representation provides a more interpretable understanding of how input features influence the outputs of a single forward pass.

#### Perplexity (per word)

Perplexity is a fundamental metric used to measure the difficulty a statistical model has in making a prediction^41^. It quantifies the model’s ability to predict a sequence of words by computing the exponential of the average negative log-likelihood of the predicted probabilities. A lower perplexity indicates a model that is more confident and accurate in its predictions, while higher perplexity suggests greater uncertainty. The per-word version normalizes this value by the number of tokens, providing a direct measure of predictive difficulty on a per-token basis. Formally, perplexity is defined as:

4 $$PPL=\exp \left(-\frac{1}{N}{\sum }_{i=1}^{N}\log p({w}_{i} \, | \, {w}_{ < i})\right)$$

where *N* denotes the number of tokens in the test corpus, *w*_*i*_ is the *i*-th word, and *p*(*w*_*i*_∣*w*_<*i*_) represents the model’s conditional probability of generating word *w*_*i*_ given the preceding context. This measure provides an interpretable indication of how effectively the model generalizes to unseen data, reflecting both fluency and predictive calibration.

#### Elo ranking

The Elo ranking system, originally developed for chess, was employed to evaluate the relative performance of different agents in pairwise comparisons. Each agent’s rating was updated based on the outcomes of these comparisons, reflecting their relative skill levels. The Elo ranking formula is given by:

5 $${R}^{{\prime} }=R+k\cdot (S-E)$$

where ${R}^{{\prime} }$ is the updated Elo rating, *R* is the current rating, *k* is the update factor, *S* is the score achieved in the match, and *E* is the expected score, calculated as:

6 $$E=\frac{1}{1+1{0}^{({R}_{opp}-R)/400}}$$

Here, *R*_*opp*_ represents the opponent’s rating. For our experiments, we set *k* = 16 and initialized all agent ratings at 1200. The score *S* was assigned as *S* = 1 for a win and *S* = 0.5 for both agents in the case of a draw. Pair-wise comparisons were conducted with Prompt S4 (supplementary files) as our judge.

##### Experimental protocol

For each question, a random pair of models was selected, and their responses were blindly evaluated by a third-party LLM judge for correctness and contextual relevance. The outcome of each comparison was recorded as a win, loss, or draw, contributing to an Elo score update. This process was repeated until all questions had been evaluated once, constituting a single round. The entire protocol was then repeated three times with new random pairings per question, and the final Elo rankings were obtained by averaging the results across all rounds.

##### Human evaluation

In the novel control strategy experiments, we repeated the same pairwise comparison procedure with human experts, who independently reviewed and ranked model responses based on technical correctness. These assessments were then compared against the Elo-based LLM evaluations to validate consistency and measure alignment between human and automated judgment. Notably, both human evaluators and LLM judges were provided only with the final corrective command, rather than the full chain-of-thought reasoning, to minimize bias toward linguistic fluency and focus solely on the accuracy of the proposed actions.

## Supplementary information

Supplementary Information

Description of Additional Supplementary Files

Supplementary Files

Transparent Peer Review file

## Source data

Source Data

## Data Availability

A small sample of the data used for model training in this study has been deposited in the Hugging Face dataset repository @cemag/tl-caxton. The weights of the pre-trained CIPHER model generated in this study have been deposited in the Hugging Face model repository @cemag/cipher-printing. Prompts used for different operations are available in the project repository at https://github.com/cam-cambridge/CIPHER^42^ and in the Supplementary Information. Source data generated in this study are provided with this paper. All other data are available from the corresponding authors upon request. Source data are provided with this paper.

## Code availability

The code described here and the relevant experiment scripts are in the project’s repository: https://github.com/cam-cambridge/CIPHER^42^.
