# Supplementary material for: Hybrid reasoning for perception, explanation, and autonomous action in manufacturing
Source: Nat Commun. 2026 May 18;17:6589. doi: 10.1038/s41467-026-72378-9 (PMC13381764; doi:10.1038/s41467-026-72378-9)
Supplement: Supplementary file 1 — Supplementary Information [file 41467_2026_72378_MOESM1_ESM.pdf]

Supplementary Information

Hybrid Reasoning for Perception, Explanation, and Autonomous Action in Manufacturing

Christos Margadji[1*] and Sebastian William Pattinson[1*]

[1] Department of Engineering, University of Cambridge, Trumpington Street, Cambridge, CB2 1PZ, United Kingdom.

See Tables S1–S3 and Figures S1–S7. Additional supplementary files are also available.

Supplementary files

- `/geometries`: A collection of `.gcode` files generated by CIPHER during our primitive-based shape generation experiments. Some of the parent scripts can be found in the `test.ipynb` file.
- `/infill_types`: A collection of `.gcode` files generated by CIPHER during our infill experiments, shown in Figure S7a.
- `/optimal_settings`: The assumed optimal settings for generating process-control scenarios for our unseen process experiments, shown in Figure S6.
- `/prompts`: The collection of prompts considered and used during our study. Prompts 1–5 are cited in the paper.
- `/ShapE`: A collection of `.ply` files generated by the implicit shape generator, along with the `.gcode` files obtained from them during CIPHER's processing.

Table S1: **Extended results from training configurations E.1–E.7.** E.0 represents the pre-trained Llama 3.2 Vision-Instruct (11b) model. Error bars: standard deviation, across all averaged samples.

Exp.	Configuration				Notes	Metrics			
	⋆1	⋆2	⋆3	⋆4		Reg. MAE	Cls. Acc.	Lang. Recall	VQA CIDEr
E.0	–	–	–	–	Baseline. Out-of-box Llama 3.2 11B Vision model	n/a	n/a	0.96 ± 0.02	0.41 ± 0.43
E.1	✓	✓	–	–	Vanilla finetuning of the language and vision components of the baseline model.	82.92 ± 71.83	29.77 ± 2.03	0.01 ± 0.01	0.00 ± 0.00
E.2	–	–	✓	–	The baseline model is integrated with a process expert and only a small projection layer is trained while vision and language modules are frozen.	17.52 ± 28.89	88.25 ± 1.46	0.90 ± 0.03	0.38 ± 0.49
E.3	✓	–	✓	–	The baseline model is integrated with a process expert and the language component is fine-tuned while the vision component remains frozen.	17.30 ± 29.33	87.28 ± 1.48	0.04 ± 0.03	0.00 ± 0.00
E.4	–	✓	✓	–	The baseline model is integrated with a process expert and the vision component is fine-tuned while the langugage component remains frozen.	17.62 ± 30.04	88.59 ± 1.47	0.88 ± 0.03	0.00 ± 0.00
E.5	✓	✓	✓	–	Vanilla finetuning of the language and vision components plus the model is integrated with a process expert.	17.78 ± 29.95	87.57 ± 1.42	0.00 ± 0.00	0.00 ± 0.00
E.6	–	–	–	✓	Baseline model fine-tuned with low rank adaptation.	61.95 ± 52.92	10.15 ± 1.41	0.86 ± 0.04	0.12 ± 0.13
E.7	–	–	✓	✓	Baseline model integrated with process expert and fine-tuned with low rank adaptation.	19.07 ± 31.13	79.46 ± 1.79	0.88 ± 0.03	0.24 ± 0.21

⋆1: trainable language module, ⋆2: trainable vision module, ⋆3: Process expert; ⋆4: low-rank adaptation enabled. ✓ indicates the component is enabled

Table S2: **Elo ratings for question answering and reasoning.** For all rankings we conducted three rounds of assessments, each round is a pair-wise comparison between random candidates. A higher Elo rating indicates better relative response quality.

(a) Correctness in question answering.

	Ours	Ours+RGA	Llama-3.2	GPT-4o mini	GPT-4o
Round 1	1093	1197	1174	1253	1283
Round 2	1066	1238	1171	1264	1261
Round 3	1099	1171	1179	1246	1305

(b) Relevance in question answering.

	Ours	Ours+RGA	Llama-3.2	GPT-4o mini	GPT-4o
Round 1	1099	1219	1143	1308	1232
Round 2	1129	1170	1194	1266	1242
Round 3	1092	1157	1243	1258	1251

(c) Physics-informed chain-of-thought reasoning.

	Ours	Ours(+RGA)	Ours(+codebook)	Llama-3.2	GPT-4o mini	GPT-4o
Round 1	1139	1220	1232	1141	1211	1257
Round 2	1131	1210	1231	1173	1224	1231
Round 3	1106	1240	1250	1150	1218	1236

Table S3: **Hyperparameters used to train our CIPHER framework.**

(a) Global

Parameter	Value
Num Train Epochs	1
Train Batch size	4
Gradient Steps	8
Learning rate (Llama)	2e-4
Learning rate (v. expert)	1e-4
Max. gradient norm.	0.3
Warmup ratio	0.03
Max Length	2014

(b) LoRA (applied on VLA)

Parameter	Value
alpha	16
dropout	0.05
Rank	8
Target modules	queries,keys
Quantization	True, 4-bit

(c) Expert

Parameter	Value
Warmup batch size	16
Learning Rate	1e-4
Scheduler	Step
Warmup iterations	500,000

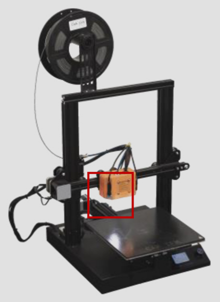

User: What do you see?

GPT-4o: This is a **3D printer.**
Specifically, it looks like a **Cartesian-style FDM (Fused Deposition Modeling)** 3D printer — a very common type used by hobbyists and professionals alike.
Key features visible in the image:
[…].
It's used for creating 3D objects from digital models (STL or G-code files).

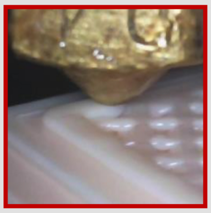

User: What do you see?

GPT-4o: The image appears to show the tip of a 3D printer nozzle extruding material—[…].

User: : What is the flowrate value?

GPT-4o: I can't determine the **exact flow rate** (in mm^3/s or similar) from this image alone — there's not enough quantitative information (like extrusion speed, filament diameter, nozzle diameter, or print settings).

Fig. S1: **Responses on high-level and low-level manufacturing scenarios.** In the latter, macro inputs are given and granular information is requested, leading to high model uncertainty.

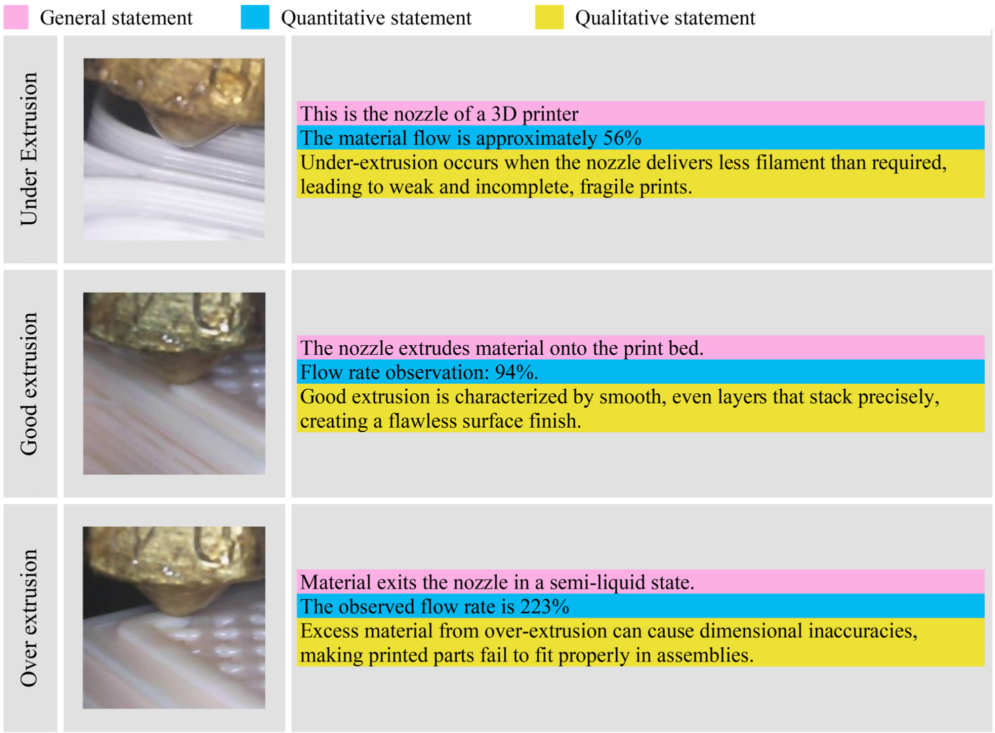

Fig. S2: **Example image-caption pairs used to train the CIPHER.** Captions are divided into three components: general, quantitative, and qualitative. Based on the continuous flow rate label, samples are categorized as over-extrusion, under-extrusion, or good extrusion.

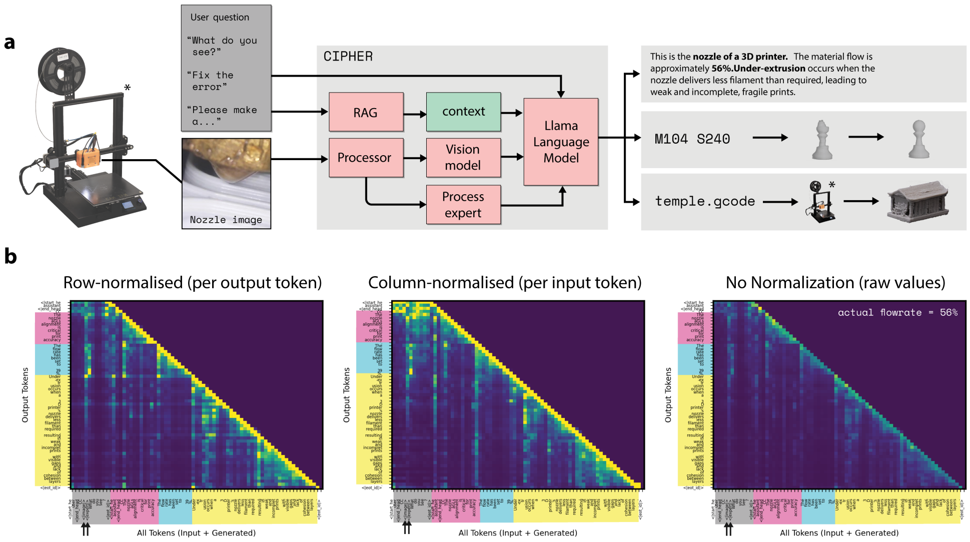

Fig. S3: **Inference details.** (a) The end-to-end inference pipeline for CIPHER where vision and textual inputs are converted into actionable insights. (b) Row-normalized, Column-normalized and Non-Normalized attention matrices from the same inference from model E.2. The first two reveal where new tokens attend and where old tokens contribute, respectively.

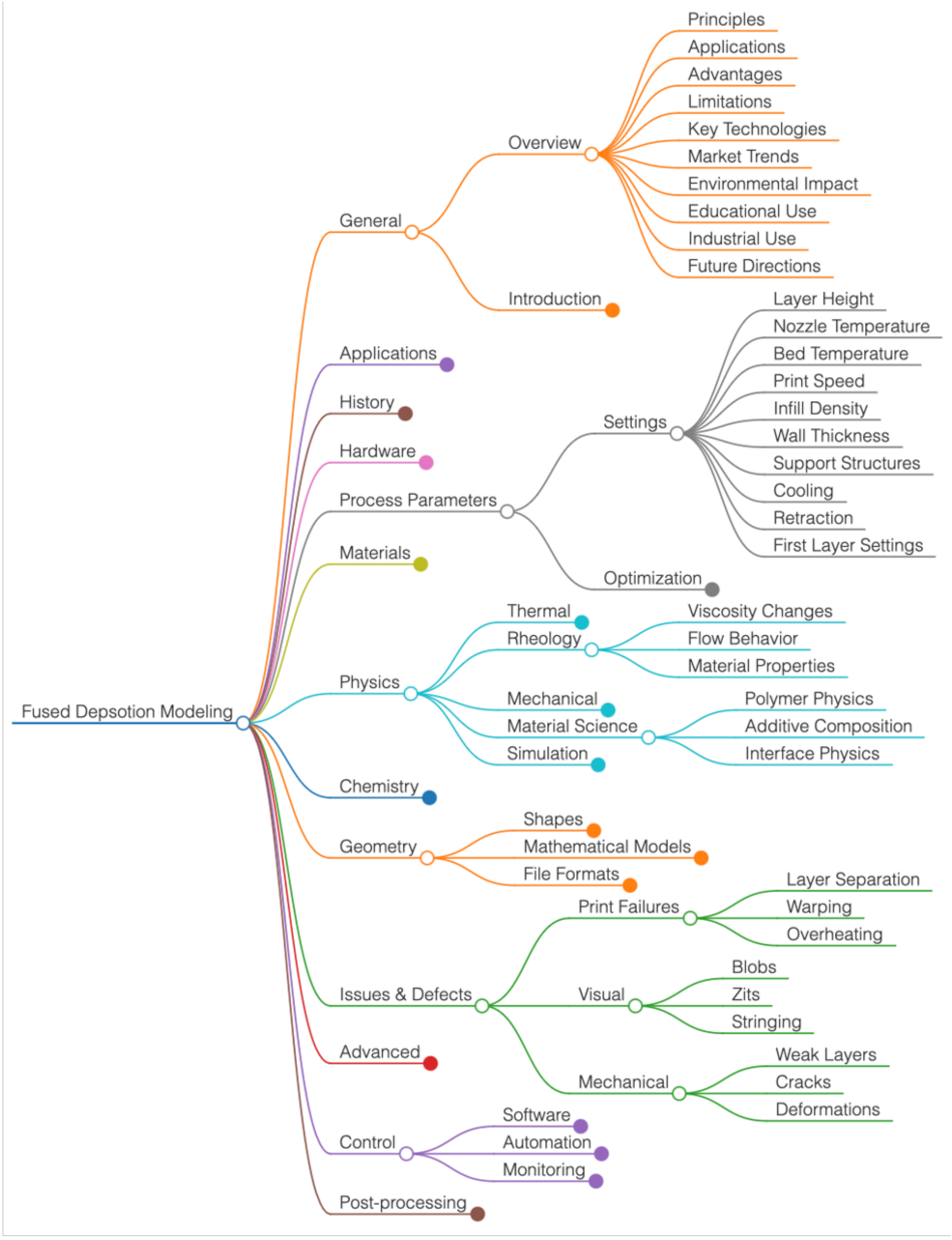

Fig. S4: **Mind map used to guide the fact generation used for retrieval-augmented generation.**

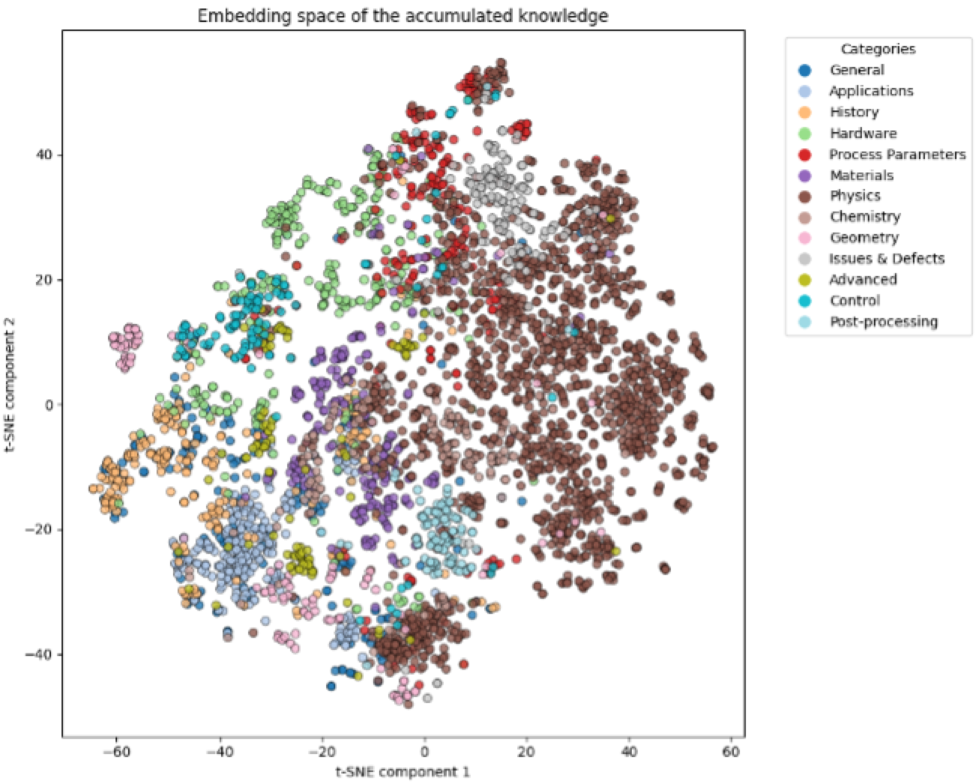

Fig. S5: **T-SNE plot of the embedding space of all gathered knowledge used for retrieval-augmented generation.**

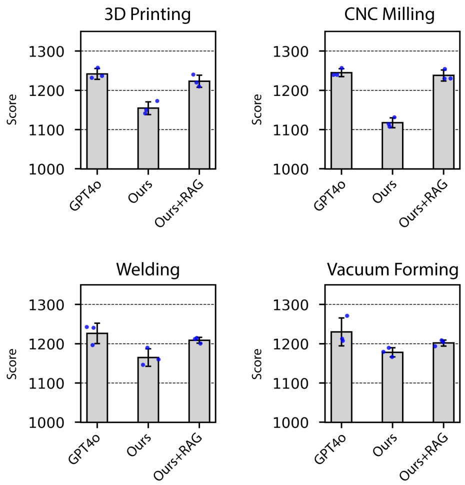

Fig. S6: **CIPHER on novel processes.** Elo ratings for control command quality in novel manufacturing processes: 3D printing, CNC Machining, Welding and Vacuum Forming. Error bars: standard deviation, 3 rounds, 100 challenges each.

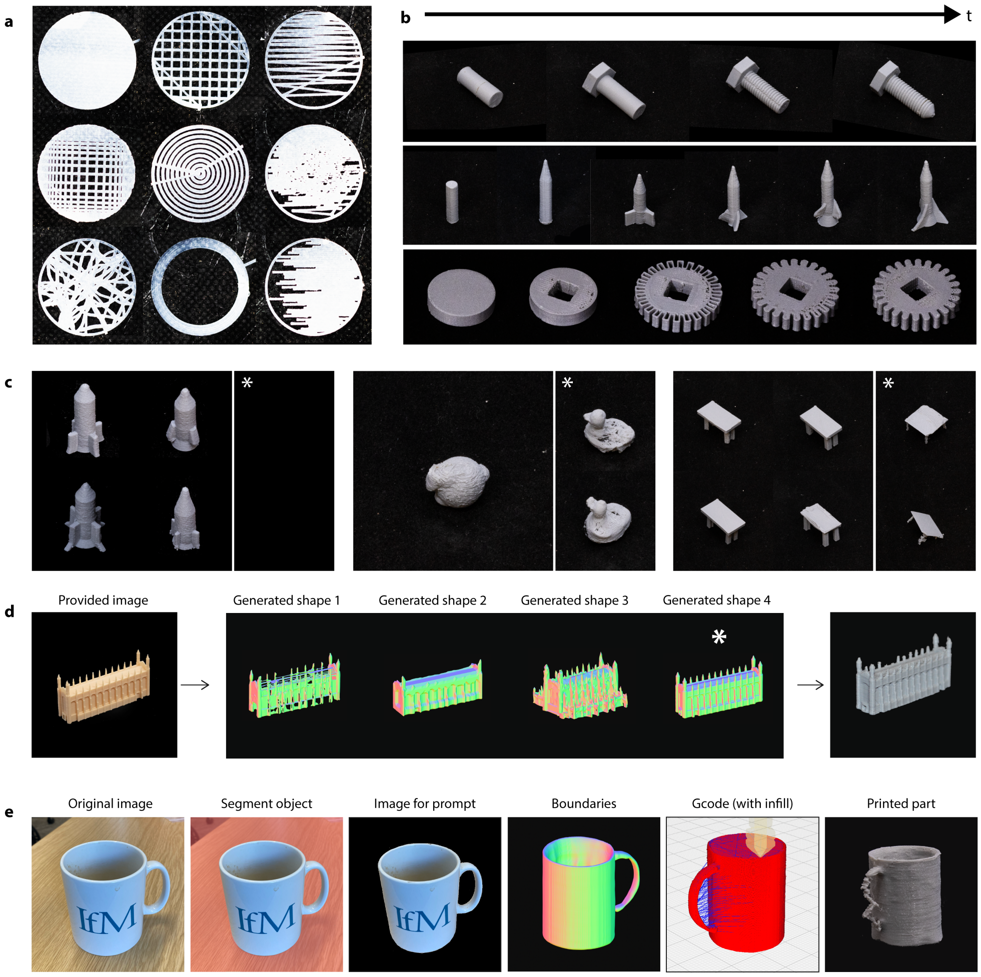

Fig. S7: **Additional experiments on geometry expert.** (a) Examples of infill patterns generated by CIPHER, including gradient infills along the toolpath direction—configurations not achievable with conventional slicers. (b) Progressive experiments where CIPHER constructed geometries from explicit plans versus ambiguous language, showing clear improvements in fidelity. (c) Repeatability tests and comparison with Shap-E (shown with *) outputs; CIPHER succeeded in cases where the shape generator failed (e.g., rocket, table) and vice versa (e.g., duck). (d) The agent proposes four candidate shapes for user selection before G-code generation and printing; * marks the chosen design. (e) End-to-end workflow from image input to 3D-printed object.
