# Supplementary material for: Hybrid reasoning for perception, explanation, and autonomous action in manufacturing
Source: Nat Commun. 2026 May 18;17:6589. doi: 10.1038/s41467-026-72378-9 (PMC13381764; doi:10.1038/s41467-026-72378-9)
Supplement: Supplementary file 2 — Description of Additional Supplementary Files [file 41467_2026_72378_MOESM2_ESM.pdf]

Description of Additional Supplementary Files

Supplementary Files:

/geometries: A collection of .gcode files generated by CIPHER during our primitive-based shape generation experiments. Some of the parent scripts can be found in the test.ipynb file.

/infill_types: A collection of .gcode files generated by CIPHER during our different infill experiments, shown in Figure S7a.

/optimal_settings: The assumed optimal settings for generating process control scenarios for our unseen process experiments, shown in Figure S6.

/prompts: The collection of prompts considered and used during our study. Prompt 1 to 5 are cited in the paper.

/ShapE: A collection of .ply files generated by the implicit shape generator, along the .gcode files obtained from them during CIPHER's processing.
