# Supplementary figures and images for: Hybrid reasoning for perception, explanation, and autonomous action in manufacturing
Source: Nat Commun. 2026 May 18;17:6589. doi: 10.1038/s41467-026-72378-9 (PMC13381764; doi:10.1038/s41467-026-72378-9)

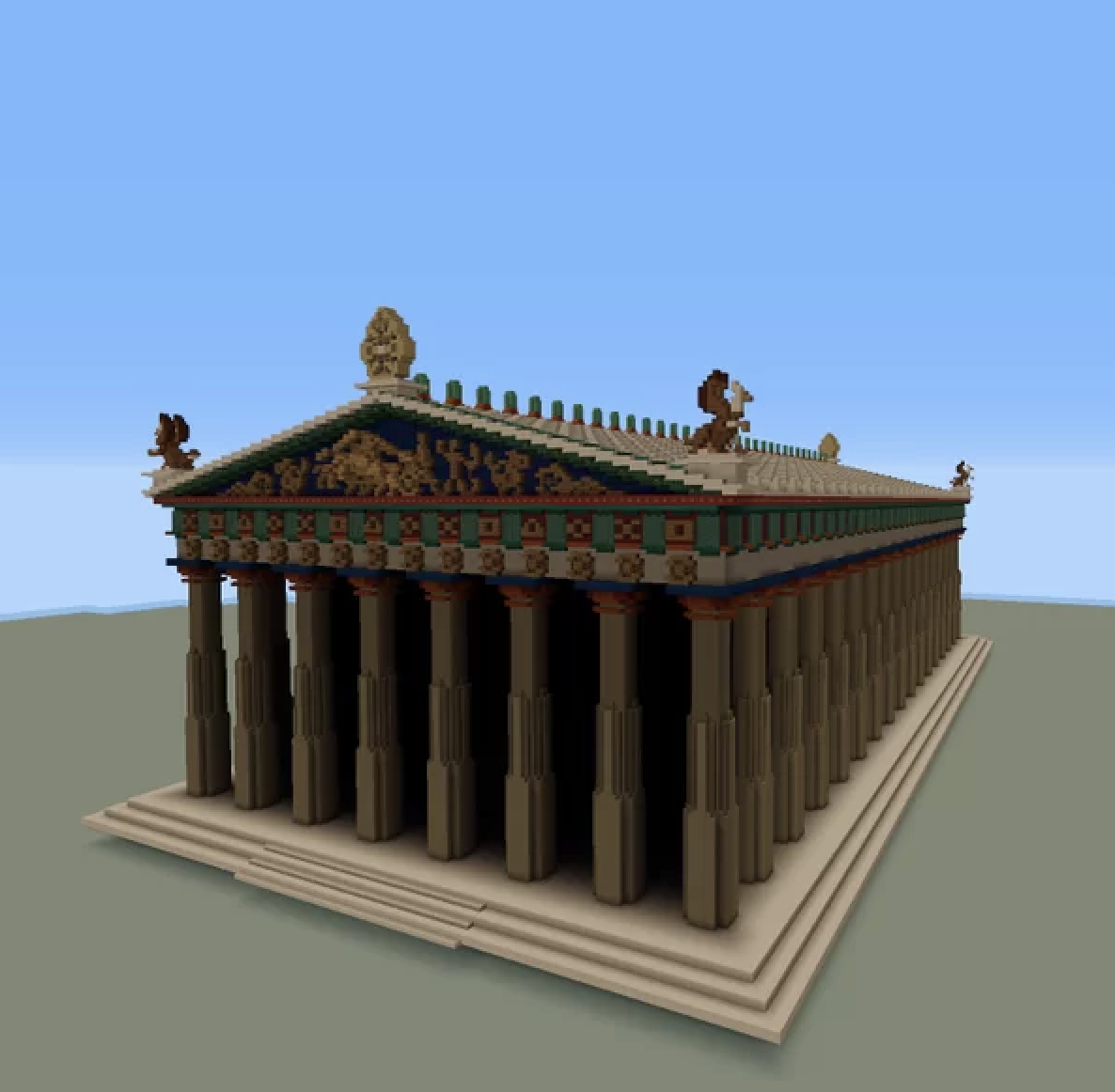

Supplement: Supplementary file 3 — Supplementary Files [file 41467_2026_72378_MOESM3_ESM.zip › supplementary-files/ShapE/temple.png]

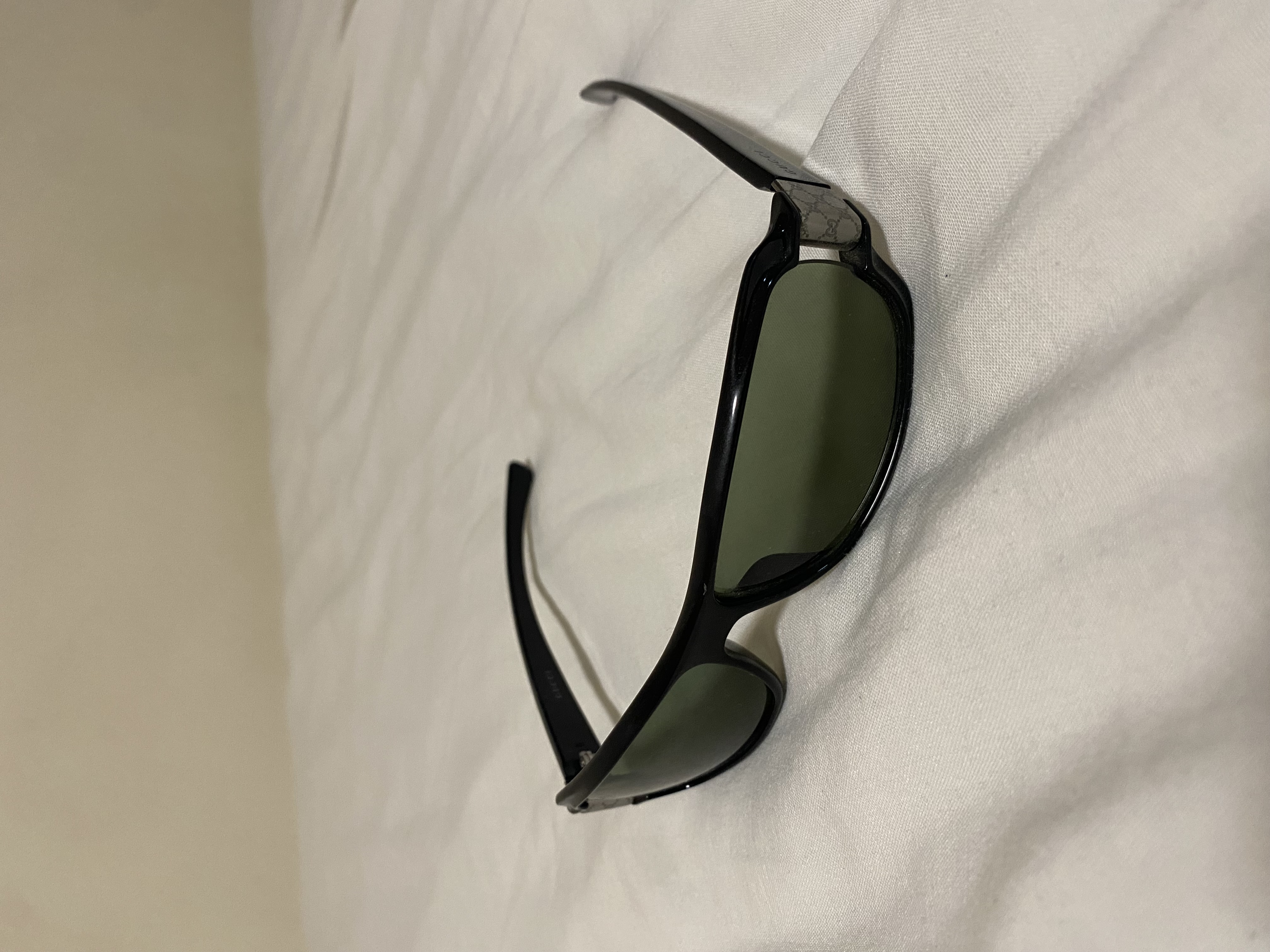

Supplement: Supplementary file 3 — Supplementary Files [file 41467_2026_72378_MOESM3_ESM.zip › supplementary-files/ShapE/IMG_9338.jpeg]

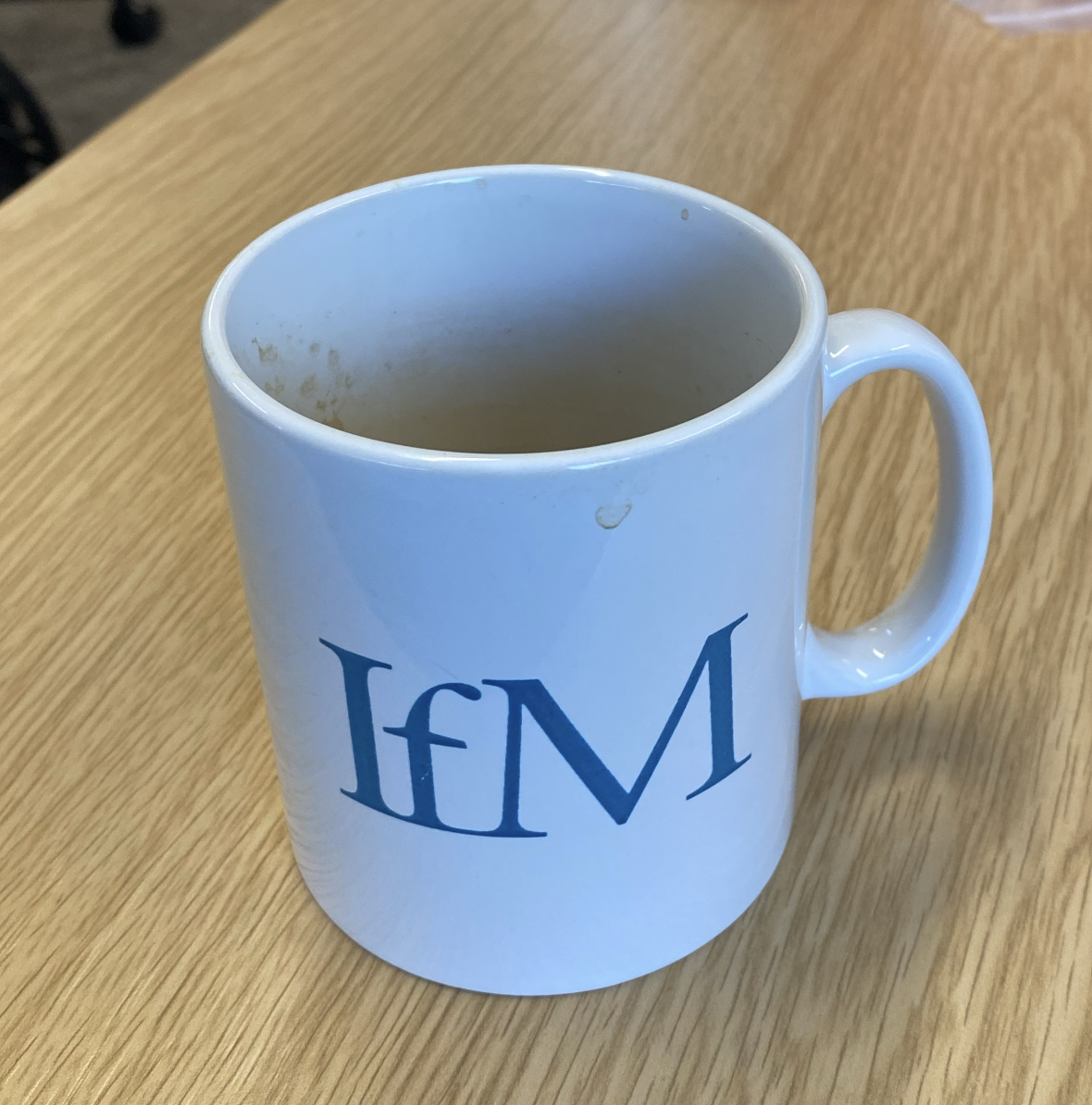

Supplement: Supplementary file 3 — Supplementary Files [file 41467_2026_72378_MOESM3_ESM.zip › supplementary-files/ShapE/cup.png]
