# Supplementary material for: Hybrid reasoning for perception, explanation, and autonomous action in manufacturing
Source: Nat Commun. 2026 May 18;17:6589. doi: 10.1038/s41467-026-72378-9 (PMC13381764; doi:10.1038/s41467-026-72378-9)
Supplement: Supplementary file 4 — Transparent Peer Review file [file 41467_2026_72378_MOESM4_ESM.pdf]

nature portfolio

Peer Review File

Hybrid Reasoning for Perception, Explanation, and Autonomous Action in Manufacturing

Corresponding Author: Dr Sebastian Pattinson

This file contains all reviewer reports in order by version, followed by all author rebuttals in order by version.

Version 0:

Reviewer comments:

Reviewer #1

(Remarks to the Author)
The authors present CIPHER, a hybrid, AI-driven tool for supporting advanced manufacturing, specifically additive manufacturing. They describe the development and application of the tool before going into details about each of its component, drawing conclusions about AI-driven manufacturing at the end.

The authors address the deployment of foundational models into domain-specific applications, a very relevant topic with significant importance. Namely, the authors explore the training and configuration of LLM and multi-modal foundational models and propose a hybrid method (using artificial narrow intelligence of VLA) while also leveraging fine tuning, prompt engineering, and RAG options. The authors demonstrate that their agent-driven framework is able to outperform baseline models against varying metrics. The ability to tailor foundational models for specific applications promises to be one of the next breakthroughs in AI adoption.

The authors do an excellent job of describing the development of their framework and its components. However, the authors seemingly chose to focus primarily on the agnostic / data science aspects of its development, notably the adjustments made during the training and manipulation of the models. This reviewer was hoping to gain additional insight into the domain considerations, and adjustments made for domain-driven applications The authors present CIPHER, a hybrid, AI-driven tool for supporting advanced manufacturing, specifically additive manufacturing. They describe the development and application of the tool before going into details about each of its component, drawing conclusions about AI-driven manufacturing at the end.

The authors address the deployment of foundational models into domain-specific applications, a very relevant topic with significant importance. Namely, the authors explore the training and configuration of LLM and multi-modal foundational models and propose a hybrid method (using artificial narrow intelligence of VLA) while also leveraging fine tuning, prompt engineering, and RAG options. The authors demonstrate that their agent-driven framework is able to outperform baseline models against varying metrics. The ability to tailor foundational models for specific applications promises to be one of the next breakthroughs in AI adoption.

The authors do an excellent job of describing the development of their framework and its components. However, the authors seemingly chose to focus primarily on the agnostic / data science aspects of its development, notably the adjustments made during the training and manipulation of the models. This reviewer was hoping to gain additional insight into the domain considerations, and adjustments made for domain-driven applications.

Specifically, the authors make it clear that there is a specific domain in mind with their manuscript, 'aiming to replicate human-like reasoning for industrial control, instantiated in a commercial-grade 3D printer." However, the scope at which this goal is to be achieved could be better articulated. Figure 1 describes their framework, noting process control and geometric components. However, the manuscript seems to focus on training for the process control components, a observation strengthened by Figures 2 and 4. Figure 6, however, brings geometric complexity into purview as well. With the range of applications addressed by the framework, how performance is consistently evaluated withing "domain-specific" applications is not entirely clear.

This manuscript would benefit from additional benchmarking details and training metrics. While figure 3 was helpful in

understanding the capabilities of the different configurations, further explanation into the implications of the various configurations would be helpful. How did different modifications relatively compare to domain-oriented results? Figure 4 was very insightful and provided insight into the effect of different configurations, additional, similar insights would strengthen the paper.

The authors provide a detailed account of their framework with potentially significant contributions. The story to be told by their manuscript, however, could be more succinct by providing additional scoping, domain details, and metric and benchmarking details. This reviewer would like to see these additional details strengthen a key message provided in the discussion, "While large foundation models have demonstrated impressive generalization
in language and vision tasks, their integration into physical domains has remained
constrained by their limited ability to represent and reason over continuous, domainspecific
parameters. CIPHER addresses this limitation through a modular architecture
that combines a vision-language transformer with a dedicated regression expert and
retrieval-augmented reasoning.......This expert
integration improves both quantitative prediction and semantic coherence between
natural language and system state, suggesting a useful pathway for combining pretrained
models with specialized modules when task precision is essential."

(Remarks on code availability)
The authors do a commendable job of making their framework and methods retrievable on github.

Reviewer #2

(Remarks to the Author)
This manuscript presents CIPHER, a hybrid vision-language-action (VLA) framework designed to support autonomous control in a manufacturing context. The authors implement the framework on a 3D printing system and claim capabilities such as context-aware reasoning, natural language explanation of control decisions, quantitative perception via a "process expert", and geometry generation from abstract prompts. The work addresses a well-recognised challenge: the application of large language models (LLMs) and vision-language models (VLMs) to domains requiring numeric precision and domain-specific knowledge, such as manufacturing.

Noteworthy Results
The integration of a vision-language model with a regression-oriented process expert and a retrieval-augmented generation (RAG) mechanism is technically competent and addresses a recognised shortcoming of general-purpose foundation models in physical control contexts. The system exhibits some emergent behaviours, including the ability to generate explanatory statements linked to observed errors and to propose corrective actions. The experimental validation within the 3D printing context, including flow rate control, error diagnosis, and geometry translation from natural language, demonstrates system-level functionality and interaction between the modules.

Originality and Significance
While the integration is novel within this specific context, the individual components - ViTs, LLMs, LoRA-based fine-tuning, regression heads, and retrieval modules - are all standard techniques. The primary innovation lies in the orchestration of these techniques for end-to-end reasoning and action in a manufacturing system. This is a respectable systems-level contribution, but not a conceptual or algorithmic one. The work does not introduce a new learning paradigm, model architecture, or theoretical insight into embodied reasoning or hybrid AI.

The impact is therefore contingent on generalisability. However, the framework is only evaluated in a constrained domain (fused deposition modelling), and it remains unclear whether the approach is transferable to other processes or physical systems. No principles or abstractions are offered to guide such transfer, which limits the breadth of the contribution. Without broader validation or theoretical framing, the work remains an interesting case study rather than a platform or methodology of general relevance.

Support for Claims and Conclusions
The claims of context-sensitive reasoning and emergent behaviour are supported to some extent by the qualitative outputs and the use of chain-of-thought generation. However, these are evaluated primarily using LLM-based pairwise comparison metrics (Elo rankings from GPT-4o), which are indirect and may be biased towards linguistic fluency rather than technical correctness. There is no evidence of expert human evaluation of these explanations, nor of their utility to end-users in practice.
The claim that CIPHER bridges the gap between traditional controllers and foundation models by offering precise and interpretable control is partially substantiated, particularly for the flow-rate regression and G-code generation tasks. However, some of the conclusions - for example, regarding explainability, generalisation, and trustworthiness in high-stakes settings - are somewhat overstated relative to the limited scope and controlled nature of the experiments.

Methodological Soundness
The methods used are appropriate for the stated goals. The inclusion of ablation studies, classification and regression benchmarks, and exploration of catastrophic forgetting is commendable. The use of parameter-efficient fine-tuning (LoRA) and minimal modification to pretrained components is well motivated and aligns with best practice.

However, the system is highly tailored to a single setup, and several components (e.g. the knowledge base used in retrieval, the geometry module, and the process expert) rely on domain-specific assumptions or curated templates. The approach is therefore not "foundational" in any general sense. The inclusion of a geometry expert is interesting, but its actual geometric reasoning capabilities appear rudimentary, relying on scripted compositions and an external implicit shape generator without verification of manufacturability.

Transparency and Reproducibility
While a GitHub repository is provided, the code is sparse and includes hardcoded local paths, which limit its portability and immediate usability. Core components of the system – particularly the RAG module, knowledge base construction, and full prompt logic – are not included or are only schematically represented. The geometry module in particular is only partially represented. Without access to the training data or a reproducible pipeline for the presented experiments, independent verification is not currently feasible. This weakens confidence in the robustness of the reported results and reduces the utility of the work to the broader research community.
The Methods section is generally detailed and well organised, and does describe the training and architecture components adequately for conceptual understanding. However, additional information on failure cases, parameter sensitivity, and data limitations would help clarify the boundaries of the system's capabilities.

Overall Assessment
This manuscript presents an ambitious attempt to combine perception, quantitative modelling, and reasoning in a single autonomous control agent. It demonstrates respectable engineering, clear articulation of motivation, and systematic internal validation. However, the work is limited by its narrow domain scope, lack of proven generalisation, reliance on standard components, and incomplete code sharing. While it may be of interest to researchers in intelligent manufacturing systems or applied embodied AI, its broader scientific contribution remains modest. Clarification of the limitations, improvement of the codebase, and more convincing demonstrations of generalisability would be required to elevate this to a more foundational contribution.

Reviewed by
Aydin Nassehi

(Remarks on code availability)
The authors provide a GitHub repository (https://github.com/cam-cambridge/CIPHER) intended to support reproducibility and transparency. While this is commendable in principle, the current state of the repository does not provide sufficient resources to enable independent replication of the results described in the manuscript.

The codebase is relatively sparse given the complexity of the system outlined. Key components such as the retrieval-augmented generation (RAG) pipeline, the knowledge base construction process, chain-of-thought scaffolding, and the geometry expert's filtering and composition logic are either omitted or represented schematically. In addition, the repository contains hardcoded local file paths that would fail on external systems unless manually modified, which limits portability and usability. There is no clear example or baseline script demonstrating inference or evaluation on even a minimal dataset.

Furthermore, the dataset used for training and evaluation (images, process parameters, captions) is not available, and there are no surrogate examples or mock data files provided to illustrate the expected data format. As a result, users cannot test the system end-to-end or verify any of the reported numerical results without substantial reconstruction effort.

The README is concise and outlines the purpose of the project and some installation steps, but it lacks detailed documentation of module functions, dependencies, or usage examples. While the code may serve as a conceptual reference for researchers interested in combining vision-language models with domain-specific modules, it currently falls short of providing a reusable or verifiable implementation.

Although the intent to share code is appreciated, the present state of the repository does not meet the standards of reproducibility expected. The authors are encouraged to expand the repository to include:

* Fully functional, path-agnostic scripts for training and inference;

* Sample data and prompts;

* Documentation clarifying the roles and usage of each module.

Such improvements would enhance the value of the work to the community and support the transparency the journal advocates.

Reviewer #3

(Remarks to the Author)
I co-reviewed this manuscript with one of the reviewers who provided the listed reports. This is part of the Nature Communications initiative to facilitate training in peer review and to provide appropriate recognition for Early Career Researchers who co-review manuscripts.

(Remarks on code availability)

Reviewer #4

(Remarks to the Author)
The foundation of the CIPHER framework is a vision-language-action (VLA) model, a class of systems designed to unify perception, language understanding, and physical action. The authors use the Llama-3.2-11B-Vision architecture, which comprises a vision encoder, a projection layer, and a large language model (LLM).

The primary vision component of this module is a Vision Transformer (ViT-H/14), a powerful, general-purpose image encoder. Its function within CIPHER is to perform general-purpose perception: it takes the raw pixels of the input image (a view of the 3D printer nozzle) and transforms them into a rich, high-dimensional feature representation. This representation captures a qualitative, holistic understanding of the visual scene, which is then passed to the LLM. This is the standard operational principle of VLA models, where the vision module provides the contextual "what I see" information to the reasoning core of the system.

However, the authors explicitly identify a critical limitation of this generalist approach. They state that foundation models like VLMs "exhibit weaker regression capabilities" and struggle with the "quantitative precision demanded by engineering applications". This is not just a theoretical concern; their own ablation studies confirm it. When they attempted to fine-tune the base VLA model to predict the numerical flow rate directly (experiment E1), the model failed to learn the quantitative task, with the mean absolute error (MAE) remaining high and unconverged. This failure highlights a fundamental mismatch: the classification-based loss functions and next-token prediction mechanisms that make LLMs powerful reasoners are ill-suited for precise, continuous-value regression. This establishes the critical need for a different architectural solution.

The methodology is sound and meets the expected standards for research in this field.
The methodology is generally well explained, but a supplementary step-by-step flowchart illustrating the data flow from input image to final LLM prompt would be recommended. Moreover, it would be helpful to explicitly naming the "publicly available multi-modal foundation models" used for initial benchmarking to enhance transparency and allow for better contextualization of the findings.

A further minor recommendation is to clearly state at the beginning that the work is about polymer 3d Printing and which technology.

In conclusion, the paper presents a promising approach for hybrid reasoning in industrial AI.

(Remarks on code availability)

Reviewer #5

(Remarks to the Author)
The work describes a method to control a Fused Filament Fabrication (FFF) process using a novel hybrid approach called CIPHER (a VLA framework). This approach is based on different AI models designed to assess and improve manufacturing precision by autonomously generating coherent g-code commands. The deposition quality is monitored by a camera mounted on the nozzle. When necessary, the process is primarily adjusted by modulating the flow rate through modifications to the feed rate or temperature.
The topic addressed in this manuscript is of significant interest to the scientific community. While the underlying framework appears to be established, its application to the field of Additive Manufacturing is novel.

The paper is well-organized and easy to follow; the figures and tables are clear and concise.

Here are some suggestions to improve the work.

1 - If possible, better describe the various "E" AI architectures/models in the text.

2 - The authors should discuss the method's applicability to other manufacturing processes (both AM and non-AM) and the possible modifications.

3 - Please provide a more detailed description of the experimental setup, including a picture for clarity.

4 - Can the system generate toolpaths for objects with functionally graded properties (e.g., material or extrusion gradients)?

5 - Is there repeatability in the G-code creation when given the same prompt?

6 - Please include a sample of a generated g-code to illustrate the output.

7 - Does the generated g-code include infill strategies or only the external toolpaths?

8 - It would be helpful to include a figure comparing a standard part printed with the proposed method against one from a conventional setup to visually demonstrate its advantages.

9 - The authors should include a basic comparison of the performance of the generation method against other relevant approaches described in the literature or add references.

Some other minor observations:

1 - The authors should consider selecting an alternative to the yellow color used in the figures, as it has poor contrast and is not clearly visible on a white background.

2 - For improved readability, please consider inverting the top-to-bottom order of the elements in Figure 2c.

3 - The authors are advised to proofread the text within all figures for spelling errors. For instance, "lossses" in the legend of Figure 2a appears to be a typo.

4 - There seems to be an incomplete sentence or a typographical error in the main text. In Section 2.4.1, line 11, the sentence begins "For context, the command M221 controls the system's This experiment…", which needs correction.

(Remarks on code availability)

Version 1:

Reviewer comments:

Reviewer #1

(Remarks to the Author)
Overall the authors did a commendable job of addressing this reviewer's concerns, particularly when addressing each comment individually.

After reading the other reviews, reviewer 2 did a nice job of articulating some of my observations (reviewer 1), namely a need to emphasize the novelty. This reviewer believes that that novelty comes in the domain integration, both what is done and how it is done. The reviewers seemed to acknowledge this in the response to individual comments, however the edits to the document were less direct.

This reviewer believes additional clarifications could be made in regards to domain contextualization, but these comments are recommendations.
-Paul Witherell

(Remarks on code availability)
I've reviewed only at a high level. Previous reviews noted some hardcoded locations that have since been updated.

Reviewer #2

(Remarks to the Author)
This revised manuscript presents a substantially expanded and technically more developed version of the original submission. The authors have clearly invested significant effort in addressing reviewer concerns related to experimental validation, reproducibility, and empirical scope. Several additions represent substantive improvements to the paper, particularly from a systems and engineering perspective.

Noteworthy results
The most notable improvements are:
- The release of a usable codebase and training data, which meaningfully improves transparency and supports reproducibility.
- The inclusion of a human evaluation study for control-command quality, addressing concerns about bias in LLM-based judging.
- Additional experiments demonstrating application of the framework to multiple manufacturing processes (machining, welding, vacuum forming), which strengthen claims of architectural portability.
- Clearer experimental analysis of perception accuracy, uncertainty, and catastrophic forgetting.

Taken together, these additions establish the work as a carefully engineered systems demonstration of how vision-language models can be augmented with regression-based perception and retrieval mechanisms for manufacturing tasks.

Significance and relation to prior work
The work will be of interest to researchers in intelligent manufacturing systems, applied embodied AI, and hybrid AI

architectures. Its primary contribution lies in system integration and empirical demonstration, rather than in primary conceptual or algorithmic novelty.

The individual components employed (VLMs, regression heads, RAG, LoRA-based fine-tuning) are all largely well established. The novelty arises from their orchestration within a single pipeline applied to manufacturing control. This positioning is reasonable, but the manuscript would benefit from framing itself more explicitly as a systems-level contribution, rather than implying foundational advances in reasoning or physical intelligence.

Support for claims and conclusions
While the experimental results now convincingly support claims about practical functionality, several higher-level claims remain not yet fully substantiated.

In particular, the manuscript continues to describe the system as performing physics-informed or expert-like reasoning. However, the evidence presented does not yet clearly distinguish between:
- genuine causal or physical reasoning, and
- retrieval-driven pattern completion grounded in curated textual knowledge.

Examples such as temperature-viscosity adjustments are plausible and useful, but could also plausibly arise from retrieved heuristics rather than explicit understanding of physical relationships. The system does not currently demonstrate formal causal models, interventional reasoning, prediction of downstream effects, or temporal reasoning over process evolution.

As such, claims relating to physics-informed reasoning, industrial readiness, and expert-level understanding would benefit from further moderation relative to the evidence provided.

Interpretation and conceptual limitations
A central concern from the original review remains only partially addressed. While the authors correctly note that manufacturing relies on domain-specific knowledge, the form of knowledge representation used here remains largely informal and unstructured:
- Manufacturing knowledge is treated as isolated text snippets retrieved via cosine similarity.
- There is no explicit semantic structure, ontology, causal graph, or systematic parameter coupling.
- The system is not grounded in established manufacturing frameworks, standards, or quality models.

This differs from how experienced manufacturing engineers typically reason in practice, who rely on formal process models, coupled parameter interactions, constraints, and standards-driven quality frameworks.

The additional experiments demonstrate broader applicability of the approach, but they do not fully resolve this underlying limitation. Generalisation here reflects portability of a systems template, not principled abstraction of manufacturing reasoning.

Methodology and reproducibility
The methodology is technically sound and generally well executed. The expanded Methods section, ablation studies, and evaluation protocols meet expected standards in the field.

With the release of code, data samples, and pretrained models, the work is now largely reproducible in principle. Some elements of the full pipeline, particularly knowledge-base construction and prompt scaffolding, still rely on implicit design choices, but this is now clearly documented.

Overall assessment
The manuscript has evolved into a well-developed engineering contribution demonstrating what can be achieved with current vision-language models when augmented with domain-specific perception and retrieval in controlled manufacturing settings.

However, the conceptual limitations identified in the original review remain. To align claims with evidence, the paper would benefit from:

- Explicitly framing the system as a proof-of-concept or systems demonstration rather than a foundational advance.
- Substantially moderating claims of physics-informed or expert-like reasoning.
- Clearly discussing what would be required for manufacturing-grade reasoning, including formal knowledge representation, causal modelling, standards integration, temporal reasoning, and uncertainty quantification.

With these clarifications, the work would stand as a valuable and appropriately scoped contribution that delineates both the promise and current limitations of foundation models in manufacturing control.

Aydin Nassehi

(Remarks on code availability)
The codebase has been substantially improved compared to the original submission and is now organised, readable, and suitable for inspection by the research community. A clear repository structure, accompanying README, example scripts, prompt files, and geometry artefacts are provided, and obvious portability issues such as hard-coded local paths appear to

have been addressed.

The released code, together with the associated data and pretrained models, supports understanding of the system design and enables partial reproduction of key components and demonstrations. While the repository is not a fully end-to-end, push-button reproduction of all experiments, it is a usable research artefact that allows independent inspection, execution of example workflows, and exploration of the proposed framework.

The code availability now meets a reasonable standard for a complex research system and represents a meaningful improvement in transparency and reproducibility relative to the initial version.

Reviewer #3

(Remarks to the Author)
I co-reviewed this manuscript with one of the reviewers who provided the listed reports. This is part of the Nature Communications initiative to facilitate training in peer review and to provide appropriate recognition for Early Career Researchers who co-review manuscripts.

(Remarks on code availability)
The authors have made genuine improvements: refactored code, removed hardcoded paths, provided sample data and a pre-trained model on Hugging Face. The README now includes dependencies and a script table.
However, I haven't attempted installation myself. Based on inspection, the repository appears functional for researchers familiar with VLM pipelines but lacks worked end-to-end examples. The RAG knowledge base construction could be better documented.
In summary: adequate for academic reproducibility, though not immediately accessible to the broader community. Sufficient for motivated researchers with appropriate expertise to replicate the work.

Reviewer #4

(Remarks to the Author)
The authors have adequately addressed my comments.

(Remarks on code availability)

Open Access This Peer Review File is licensed under a Creative Commons Attribution 4.0 International License, which permits use, sharing, adaptation, distribution and reproduction in any medium or format, as long as you give appropriate credit to the original author(s) and the source, provide a link to the Creative Commons license, and indicate if changes were made.
In cases where reviewers are anonymous, credit should be given to 'Anonymous Referee' and the source.
The images or other third party material in this Peer Review File are included in the article's Creative Commons license, unless indicated otherwise in a credit line to the material. If material is not included in the article's Creative Commons license and your intended use is not permitted by statutory regulation or exceeds the permitted use, you will need to obtain

permission directly from the copyright holder.
To view a copy of this license, visit https://creativecommons.org/licenses/by/4.0/

Point-by-Point response to reviewers of Hybrid Reasoning for Perception, Explanation, and Autonomous Action in Manufacturing

Reviewer #1

The authors present CIPHER, a hybrid, AI-driven tool for supporting advanced manufacturing, specifically additive manufacturing. They describe the development and application of the tool before going into details about each of its component, drawing conclusions about AI-driven manufacturing at the end.

The authors address the deployment of foundational models into domain-specific applications, a very relevant topic with significant importance. Namely, the authors explore the training and configuration of LLM and multi-modal foundational models and propose a hybrid method (using artificial narrow intelligence of VLA) while also leveraging fine tuning, prompt engineering, and RAG options. The authors demonstrate that their agent-driven framework is able to outperform baseline models against varying metrics. The ability to tailor foundational models for specific applications promises to be one of the next breakthroughs in AI adoption.

The authors do an excellent job of describing the development of their framework and its components. However, the authors seemingly chose to focus primarily on the agnostic / data science aspects of its development, notably the adjustments made during the training and manipulation of the models.

Comment 1.1: This reviewer was hoping to gain additional insight into the domain considerations, and adjustments made for domain-driven applications

Author response*: Thank you for this valuable comment. We agree that the manuscript currently emphasizes the AI aspects and that our domain-driven choices should be clearer. In the revision, we now explicitly describe why we focus on specific process parameters and how this matches the way process engineers reason about FDM processes (pg. 11). We also clarify that the sensor placement, data augmentations, caption design, and RAG corpus are all derived from realistic FDM process parameters, defect modes, and terminology, rather than generic vision tasks, and that the agent's action space is restricted to valid, safety- and process-aware G-code commands, with a brief discussion of how this would transfer to other manufacturing processes (Section 4.1, pg. 15). Finally, we have clarified why specific experiments were chosen, what their results imply for the manufacturing domain, and we added a short limitations paragraph and additional reflections to make these domain considerations more explicit (throughout Results section, pg. 4 and Discussion section, pg. 15).*

The authors present CIPHER, a hybrid, AI-driven tool for supporting advanced manufacturing, specifically additive manufacturing. They describe the development and application of the tool before going into details about each of its component, drawing conclusions about AI-driven manufacturing at the end.

The authors address the deployment of foundational models into domain-specific applications, a very relevant topic with significant importance. Namely, the authors explore the training and configuration of LLM and multi-modal foundational models and propose a hybrid method (using artificial narrow intelligence of VLA) while also leveraging fine tuning, prompt engineering, and RAG options. The authors demonstrate that their agent-driven framework is able to outperform baseline models against varying metrics. The ability to tailor foundational models for specific

applications promises to be one of the next breakthroughs in AI adoption.

The authors do an excellent job of describing the development of their framework and its components. However, the authors seemingly chose to focus primarily on the agnostic / data science aspects of its development, notably the adjustments made during the training and manipulation of the models.

Comment 1.2: This reviewer was hoping to gain additional insight into the domain considerations, and adjustments made for domain-driven applications.

Author response: *This is identical to comment 1.1 and hence we have responded to this in our response to comment 1.1.*

Comment 1.3: Specifically, the authors make it clear that there is a specific domain in mind with their manuscript, 'aiming to replicate human-like reasoning for industrial control, instantiated in a commercial-grade 3D printer." However, the scope at which this goal is to be achieved could be better articulated.

Authors response: *We agree that the scope could have been more explicitly stated and now include separate paragraphs with our scope at the start of our Results section (pg. 4). We also visualize our problem formulation in Extended Data Figure A3 (pg. 35).*

Comment 1.4: Figure 1 describes their framework, noting process control and geometric components. However, the manuscript seems to focus on training for the process control components, a observation strengthened by Figures 2 and 4. Figure 6, however, brings geometric complexity into purview as well. With the range of applications addressed by the framework, how performance is consistently evaluated withing "domain-specific" applications is not entirely clear.

Authors response: *The reviewer is correct that the primary focus of the work is on process control, which is consistently evaluated, not least through the introduction of the addition of performance evaluation across multiple manufacturing processes as well as human evaluation of model control responses (figs 6 and A6). In keeping with the overarching aim of our paper to show the benefits of foundation models in manufacturing, we demonstrate the ability of the CIPHER framework to produce various geometries (fig. 7). While this brings geometric complexity into purview alongside process control, the ability of CIPHER to perform a wider range of tasks than more traditional, narrow AI models is an important contribution of this work. We have added additional discussion of this into the Results section (pg. 12) to clarify this.*

Comment 1.5: This manuscript would benefit from additional benchmarking details and training metrics. While figure 3 was helpful in understanding the capabilities of the different configurations, further explanation into the implications of the various configurations would be helpful.

Authors response: *We agree that the manuscript would benefit from additional benchmarking details and training metrics. In the revision, we therefore provide more information on the training and evaluation setup (Section 4.2.4, pg. 19); expand the discussion around Figure 3 to explain the implications and trade-offs of the different configurations (Section 2.1, pg. 5); and we*

have added a Notes column to Table A1 (pg. 30) to describe configurations E.0–E.7 in more detail and clarify how they differ.

Comment 1.6: How did different modifications relatively compare to domain-oriented results? Figure 4 was very insightful and provided insight into the effect of different configurations, additional, similar insights would strengthen the paper.

Authors response: *We agree and have now added more specific comments relating to the domain implications of the various results that we present throughout the entire Results section. We also conducted additional uncertainty experiments and show these results in a new figure (fig. 5). These provide additional insights into the inner workings of the different configurations. Again, we have added new text mapping these differences into domain-related comments which in this context primarily revolve around the importance of handling uncertainty in manufacturing environments (Section 2.3, pg. 8).*

Comment 1.7: The authors provide a detailed account of their framework with potentially significant contributions. The story to be told by their manuscript, however, could be more succinct by providing additional scoping, domain details, and metric and benchmarking details. This reviewer would like to see these additional details strengthen a key message provided in the discussion, "While large foundation models have demonstrated impressive generalization in language and vision tasks, their integration into physical domains has remained constrained by their limited ability to represent and reason over continuous, domainspecific parameters. CIPHER addresses this limitation through a modular architecture that combines a vision-language transformer with a dedicated regression expert and retrieval-augmented reasoning.......This expert integration improves both quantitative prediction and semantic coherence between natural language and system state, suggesting a useful pathway for combining pretrained models with specialized modules when task precision is essential."

Authors response: *We thank the reviewer for this suggestion and accordingly have revised the opening paragraphs of the Discussion (pg. 14) to more clearly articulate that CIPHER's hybrid reasoning framework directly addresses the limitation of foundation models in representing continuous, domain-specific physical parameters. These revisions, along with the expanded domain considerations and benchmarking analyses added throughout Results (pg. 8-12), now more explicitly reinforce this central message and strengthen the overarching argument of the manuscript.*

Reviewer #1 (Remarks on code availability):
The authors do a commendable job of making their framework and methods retrievable on github.

Reviewer #2
This manuscript presents CIPHER, a hybrid vision-language-action (VLA) framework designed to support autonomous control in a manufacturing context. The authors implement the framework on a 3D printing system and claim capabilities such as context-aware reasoning, natural language explanation of control decisions, quantitative perception via a "process expert", and geometry generation from abstract prompts. The work addresses a well-recognised challenge: the application of large language models (LLMs) and vision-language models (VLMs) to domains requiring numeric precision and domain-specific knowledge, such as manufacturing.

Noteworthy Results
The integration of a vision-language model with a regression-oriented process expert and a retrieval-augmented generation (RAG) mechanism is technically competent and addresses a recognised shortcoming of general-purpose foundation models in physical control contexts. The system exhibits some emergent behaviours, including the ability to generate explanatory statements linked to observed errors and to propose corrective actions. The experimental validation within the 3D printing context, including flow rate control, error diagnosis, and geometry translation from natural language, demonstrates system-level functionality and interaction between the modules.

Comment 2.1: Originality and Significance
While the integration is novel within this specific context, the individual components - ViTs, LLMs, LoRA-based fine-tuning, regression heads, and retrieval modules - are all standard techniques. The primary innovation lies in the orchestration of these techniques for end-to-end reasoning and action in a manufacturing system. This is a respectable systems-level contribution, but not a conceptual or algorithmic one. The work does not introduce a new learning paradigm, model architecture, or theoretical insight into embodied reasoning or hybrid AI.

Authors response: *We appreciate the reviewer's assessment and agree that CIPHER does not introduce an entirely new learning paradigm or core architecture. The novelty of our work lies in how these components are combined and instantiated to automate a wide range of tasks in the manufacturing domain that would previously require human intervention. In particular, CIPHER integrates vision transformers, a regression-oriented perception module, parameter-efficient fine-tuning, and retrieval-augmented reasoning into a unified framework that supports end-to-end quantitative perception and language-based control in manufacturing.*

To better reflect this, we have revised the manuscript to emphasise that the contribution is twofold: (i) we provide a practical and effective solution to the underexplored challenge of performing reliable regression within VLM-based pipelines, and (ii) we offer empirical insights into model dynamics under different fine-tuning configurations when a perception regressor is embedded into an autoregressive transformer (Section 2.3, pg.8). Together, these results clarify the interactions between regression-based perception and transformer-based reasoning and open a concrete pathway toward stronger quantitative perception capabilities in embodied and robotic systems.

Comment 2.2: The impact is therefore contingent on generalisability. However, the framework is only evaluated in a constrained domain (fused deposition modelling), and it remains unclear whether the approach is transferable to other processes or physical systems. No principles or abstractions are offered to guide such transfer, which limits the breadth of the contribution.

Without broader validation or theoretical framing, the work remains an interesting case study rather than a platform or methodology of general relevance.
Authors response: *We thank the reviewer for raising this important point. To address the concern regarding generalisability, we have added new zero-shot experiments across machining, vacuum forming, and welding (see pg. 12 and Extended Data Fig. A6). These demonstrate that CIPHER can diagnose anomalies and propose corrective actions in previously unseen processes by leveraging its hybrid architecture and retrieval-augmented domain knowledge.*

In addition, the revised Discussion (pg. 14–15) now articulates the underlying principles that enable such transfer, namely the separation of quantitative perception from contextual reasoning, and the use of structured knowledge retrieval to ground decisions in process-specific physics. These additions clarify how the framework extends beyond a single domain and highlight the abstractions that make it adaptable to diverse manufacturing settings.

Comment 2.3: Support for Claims and Conclusions
The claims of context-sensitive reasoning and emergent behaviour are supported to some extent by the qualitative outputs and the use of chain-of-thought generation. However, these are evaluated primarily using LLM-based pairwise comparison metrics (Elo rankings from GPT-4o), which are indirect and may be biased towards linguistic fluency rather than technical correctness. There is no evidence of expert human evaluation of these explanations, nor of their utility to end-users in practice.
Authors response: *In response to this comment, we have now performed a human evaluation study and added to the paper in fig. 6c. During these experiments, several 3D printing experts were asked to conduct comparison rounds identical to the ones conducted by the LLMs, detailed in our Methods. The interface used for our study remains online at https://cipher-eval.vercel.app . The outcome of these experiments is that human responses aligned well with those of the LLM judge.*
We also agree with the reviewer that the previous Elo rankings based on GPT-4o evaluations may have been biased toward linguistic fluency rather than technical correctness, potentially compromising the significance of those results. To address this, we now include revised evaluations in fig. 6 which both the LLM judge and human experts were provided with only the proposed control commands, rather than the accompanying chain-of-thought reasoning paragraph (see pg. 11 for distinction between the two). This adjustment eliminates stylistic bias and ensures that assessments focus solely on the quality and correctness of the final control decision, which is ultimately the most relevant measure of performance.

Comment 2.4: The claim that CIPHER bridges the gap between traditional controllers and foundation models by offering precise and interpretable control is partially substantiated, particularly for the flow-rate regression and G-code generation tasks. However, some of the conclusions - for example, regarding explainability, generalisation, and trustworthiness in high-stakes settings - are somewhat overstated relative to the limited scope and controlled nature of the experiments.

Authors response: *We have revised the Discussion (pg. 14) to moderate these claims, clarifying that our demonstrations are performed in controlled settings and do not yet reflect full industrial-scale deployment. The revised text now frames these properties as promising directions suggested by our findings rather than established capabilities, ensuring that the conclusions remain proportional to the scope of our experiments.*

Methodological Soundness
The methods used are appropriate for the stated goals. The inclusion of ablation studies, classification and regression benchmarks, and exploration of catastrophic forgetting is commendable. The use of parameter-efficient fine-tuning (LoRA) and minimal modification to pretrained components is well motivated and aligns with best practice.

Comment 2.5: However, the system is highly tailored to a single setup, and several components (e.g. the knowledge base used in retrieval, the geometry module, and the process expert) rely on domain-specific assumptions or curated templates. The approach is therefore not "foundational" in any general sense. The inclusion of a geometry expert is interesting, but its actual geometric reasoning capabilities appear rudimentary, relying on scripted compositions and an external implicit shape generator without verification of manufacturability.

Authors response: *CIPHER does rely on domain-specific assumptions, which aligns with how human experts in manufacturing operate. In the agentic setting, this knowledge can be explicitly defined using the provided context.*

In our new experiments (discussed in pg.12 and fig. A6) we show that CIPHER can be very easily adapted to new processes, including machining, vacuum forming and welding, and constructing the knowledge base for these simply involves curating relevant information by any means possible. This shows that CIPHER does not rely on any specific curated templates and that it can reason over the available context in order to arrive at original conclusions and actionable insights that it has never seen before.

In this sense the geometry tasks addressed in our work are narrow yet powerful. We show that LLMs can reason effectively about shapes and that shapes which can be mathematically formulated are easier to work with. This opens new corridors for research around spatially aware LLMs, which will benefit not merely the setup we present here but also any other modern manufacturing process that relies on contemporary computer aided design (CAD) software. In addition to fig. A6, we have added extensive text to the discussion section to highlight this significance (pg. 15).

We understand there is a misunderstanding regarding the importance of the shape generator in our work. We have now made it clear that this has been implemented as an addition, and not as the main contribution of CIPHER. Though, our fallback experiments have shown a clear gap in existing technology; current shape generators lack understanding of the physical processes which are used to manufacture parts, now this work opens new corridors towards developing manufacturing constraint-aware 3D shape generators.

That said, our block-building experiments do not rely on scripted compositions either. CIPHER starts without any guidance and progressively builds a valuable skills library with reusable functions that can build any geometry a user has in mind. We have clarified this further in the text and also include our geometry scripts within the published codebase so researchers can experiment. While verification of manufacturability is not explicitly included in CIPHER, each of the objects in fig. 7 is physically 3D printed in a single-shot fashion, providing evidence of manufacturability.

Comment 2.6: Transparency and Reproducibility
While a GitHub repository is provided, the code is sparse and includes hardcoded local paths, which limit its portability and immediate usability. Core components of the system – particularly the RAG module, knowledge base construction, and full prompt logic – are not included or are only schematically represented. The geometry module in particular is only partially represented.

Without access to the training data or a reproducible pipeline for the presented experiments, independent verification is not currently feasible. This weakens confidence in the robustness of the reported results and reduces the utility of the work to the broader research community.

Authors response: *Thank you for highlighting this. We agree that the initial repository did not provide the level of portability and end-to-end reproducibility that would be ideal. We have now refactored the entire codebase to match the format of other similar research, we introduced comments and docstrings where necessary and have eliminated all hard-coded paths. We have also added missing components. We will keep monitoring our codebase for any open issues and will do our best efforts to resolve them such that we help researchers interested in reproducing our work.*

We also introduced a new directory at the root level called "scripts" which contains parameterizable shell scripts (easily executable using bash) for different tasks such as training as well as testing domain expertise, testing the geometry expert and more. In specific scripts we also add a Boolean variable called RAG; this activates / deactivates retrieval-augmented generation, and by following the code one can now access the relevant pipeline.

Data used in the paper has been uploaded to Huggingface (https://huggingface.co/datasets/cemag/tl-caxton).

We also open source a pre-trained CIPHER model (https://huggingface.co/cemag/cipher_printing).

These changes help to make the core pipeline reproducible and to enable independent inspection and extension of CIPHER.

Comment 2.7: The Methods section is generally detailed and well organised, and does describe the training and architecture components adequately for conceptual understanding. However, additional information on failure cases, parameter sensitivity, and data limitations would help clarify the boundaries of the system's capabilities.

Authors response: *We have added text describing the system's limitations in our Discussion (pg. 15).*

Comment 2.8: Overall Assessment
This manuscript presents an ambitious attempt to combine perception, quantitative modelling, and reasoning in a single autonomous control agent. It demonstrates respectable engineering, clear articulation of motivation, and systematic internal validation. However, the work is limited by its narrow domain scope, lack of proven generalisation, reliance on standard components, and incomplete code sharing. While it may be of interest to researchers in intelligent manufacturing systems or applied embodied AI, its broader scientific contribution remains modest. Clarification of the limitations, improvement of the codebase, and more convincing demonstrations of generalisability would be required to elevate this to a more foundational contribution.

Reviewed by
Aydin Nassehi

Authors Response: *We thank the reviewer for the thoughtful overall assessment and for recognising the ambition and effort behind CIPHER. We believe that the additional experiments, analyses, clarifications, and resources now provided and detailed in our responses above*

directly address the reviewer's concerns about domain breadth, generalisability, and reproducibility.

Reviewer #2 (Remarks on code availability):

Comment 2.9: The authors provide a GitHub repository (https://github.com/cam-cambridge/CIPHER) intended to support reproducibility and transparency. While this is commendable in principle, the current state of the repository does not provide sufficient resources to enable independent replication of the results described in the manuscript.

The codebase is relatively sparse given the complexity of the system outlined. Key components such as the retrieval-augmented generation (RAG) pipeline, the knowledge base construction process, chain-of-thought scaffolding, and the geometry expert's filtering and composition logic are either omitted or represented schematically. In addition, the repository contains hardcoded local file paths that would fail on external systems unless manually modified, which limits portability and usability. There is no clear example or baseline script demonstrating inference or evaluation on even a minimal dataset.

Authors Response: *We have addressed this comment in our response to comment 2.6.*

Comment 2.10 Furthermore, the dataset used for training and evaluation (images, process parameters, captions) is not available, and there are no surrogate examples or mock data files provided to illustrate the expected data format. As a result, users cannot test the system end-to-end or verify any of the reported numerical results without substantial reconstruction effort.

Authors response: *We have uploaded a dataset of 3.41k train, 330 validation and 309 test samples to hugging face (https://huggingface.co/datasets/cemag/tl-caxton).*

Despite the low cardinality of this dataset, it should be adequate to test and train the CIPHER agent. We will monitor usage of this dataset and will keep adding more samples as needed.

Within this dataset we also attach our mind map-derived knowledge base such that users can use our RAG pipelines without needing to use resource-intensive API calls to build them from scratch.

For the purposes of testing, we also uploaded a trained CIPHER agent (trained using configuration E.2) in Hugging Face (https://huggingface.co/cemag/cipher_printing). This will allow researchers without access to powerful GPUs to still run forward inference passes and replicate our results without the original computational burden.

Comment 2.11: The README is concise and outlines the purpose of the project and some installation steps, but it lacks detailed documentation of module functions, dependencies, or usage examples. While the code may serve as a conceptual reference for researchers interested in combining vision-language models with domain-specific modules, it currently falls short of providing a reusable or verifiable implementation.

Authors response: *The README file has been updated with more thorough dependencies and clear explanation of the contents of the codebase.*

We also added a table listing all available scripts and their functions, as well as their expected arguments (although these are typically defined in the first ~20 lines of the shell files.)

Comment 2.12: Although the intent to share code is appreciated, the present state of the repository does not meet the standards of reproducibility expected. The authors are encouraged to expand the repository to include:

* Fully functional, path-agnostic scripts for training and inference;

* Sample data and prompts;

* Documentation clarifying the roles and usage of each module.

Such improvements would enhance the value of the work to the community and support the transparency the journal advocates.

Authors response: *We believe that the additions outlined in our responses to the questions above have addressed these concerns.*

Reviewer #3 (Remarks to the Author):

I co-reviewed this manuscript with one of the reviewers who provided the listed reports. This is part of the Nature Communications initiative to facilitate training in peer review and to provide appropriate recognition for Early Career Researchers who co-review manuscripts.

Reviewer #4:

The foundation of the CIPHER framework is a vision-language-action (VLA) model, a class of systems designed to unify perception, language understanding, and physical action. The authors use the Llama-3.2-11B-Vision architecture, which comprises a vision encoder, a projection layer, and a large language model (LLM).

The primary vision component of this module is a Vision Transformer (ViT-H/14), a powerful, general-purpose image encoder. Its function within CIPHER is to perform general-purpose perception: it takes the raw pixels of the input image (a view of the 3D printer nozzle) and transforms them into a rich, high-dimensional feature representation. This representation captures a qualitative, holistic understanding of the visual scene, which is then passed to the LLM. This is the standard operational principle of VLA models, where the vision module provides the contextual "what I see" information to the reasoning core of the system.

However, the authors explicitly identify a critical limitation of this generalist approach. They state that foundation models like VLMs "exhibit weaker regression capabilities" and struggle with the "quantitative precision demanded by engineering applications". This is not just a theoretical concern; their own ablation studies confirm it. When they attempted to fine-tune the base VLA model to predict the numerical flow rate directly (experiment E1), the model failed to learn the quantitative task, with the mean absolute error (MAE) remaining high and unconverged. This failure highlights a fundamental mismatch: the classification-based loss functions and next-token prediction mechanisms that make LLMs powerful reasoners are ill-suited for precise, continuous-value regression. This establishes the critical need for a different architectural solution.

The methodology is sound and meets the expected standards for research in this field.

Comment 4.1 The methodology is generally well explained, but a supplementary step-by-step flowchart illustrating the data flow from input image to final LLM prompt would be recommended. Moreover, it would be helpful to explicitly naming the "publicly available multi-modal foundation models" used for initial benchmarking to enhance transparency and allow for better contextualization of the findings.

Authors response: *We have depicted the complete end-to-end pipeline, encompassing both training and inference, in fig. 2. Recognizing that that representation may lack clarity for some readers, we have supplemented it with an additional, phase-specific schematic diagram presented in fig. A3 in Extended Data.*

The reviewer correctly identified the omission of name of the multi-modal foundation model used; we have now incorporated this information into the appropriate section of the main text in pg. 4.

Comment 4.2: A further minor recommendation is to clearly state at the beginning that the work is about polymer 3d Printing and which technology.

Authors response: *In the introduction, we clarify the equivalence between additive manufacturing (AM) and the more widely recognized term 3D printing. We also specify the employed technology; fused filament fabrication (FFF). We also note, following revision, several components of CIPHER were additionally evaluated on other manufacturing processes too.*

In conclusion, the paper presents a promising approach for hybrid reasoning in industrial AI.

Reviewer #5 (Remarks to the Author):

The work describes a method to control a Fused Filament Fabrication (FFF) process using a novel hybrid approach called CIPHER (a VLA framework). This approach is based on different AI models designed to assess and improve manufacturing precision by autonomously generating coherent g-code commands. The deposition quality is monitored by a camera mounted on the nozzle. When necessary, the process is primarily adjusted by modulating the flow rate through modifications to the feed rate or temperature.
The topic addressed in this manuscript is of significant interest to the scientific community. While the underlying framework appears to be established, its application to the field of Additive Manufacturing is novel.

The paper is well-organized and easy to follow; the figures and tables are clear and concise.

Here are some suggestions to improve the work.

<u>Comment 5.1: 1 - If possible, better describe the various "E" AI architectures/models in the text.</u>

Authors response: *We have added further text to the Methods section to elaborate on the details of the "E" AI models (Section 4.2, pg. 17). We thank the reviewer for their comment.*

<u>Comment 5.2: 2 - The authors should discuss the method's applicability to other manufacturing processes (both AM and non-AM) and the possible modifications.</u>

Authors response: *This is similar to Reviewer 2 comment 2 and can be answered with the response we gave there. Briefly, we have now conducted further experiments involving other manufacturing processes, namely machining, vacuum forming and welding (fig. A6). We detail these in our Results section and describe the modifications needed in our Methods.*

<u>Comment 5.3: 3 - Please provide a more detailed description of the experimental setup, including a picture for clarity.</u>

Authors response: *We have added detail on the experimental setup to Methods (pg. 15-16). We have also added fig. A3 where we depict our additive manufacturing system equipped with a nozzle camera. Within these schematics we intend to show how nozzle images and user inputs flow through CIPHER to arrive to actionable linguistic outputs which may answer questions, fix errors and or initiate 3D prints in an end-to-end fashion.*

<u>Comment 5.4: 4 - Can the system generate toolpaths for objects with functionally graded properties (e.g., material or extrusion gradients)?</u>

Authors response: *This ability, while not core to the paper's contribution, is a good thing to highlight and we thank the reviewer for pointing it out. We have now added experimental examples of various infill types, including some with functional gradients, to fig. A7a (pg. 39) and briefly discuss our results in page 14. Exploring this ability further and building out capabilities for functionally graded properties would be a very interesting area of future research.*

<u>Comment 5.5: 5 - Is there repeatability in the G-code creation when given the same prompt?</u>

Authors response: *This is a good thing to point out and we thank the reviewer for the question. We conducted repeatability experiments and illustrate some of the results in fig. A7. These experiments show that CIPHER is not strictly deterministic when it comes to geometric solutions but conversely it comes up with different creative ideas even when presented with identical prompts. In our experiments we see this can be controlled for by modifying the sampling temperature. These experiments also revealed that CIPHER produces highly parameterizable code wherein every variable can control almost every important feature of the geometry (i.e., granular control over the rocket's height, diameter, extension of the fins and their cant angle.) We further discuss this in our Results section in pg. 14.*

<u>Comment 5.6: 6 - Please include a sample of a generated g-code to illustrate the output.</u>

Authors response: *In our supplementary material we added the G-code files used for producing the parts illustrated in figs. 7 and A7. These files were generated indirectly through Python code, which when executed produces the G-code files. We also include the corresponding Python snippets for reproducibility.*

<u>Comment 5.7: 7 - Does the generated g-code include infill strategies or only the external toolpaths?</u>

Authors response: *We have now conducted additional experiments in which the infill type is treated as a design variable rather than a fixed parameter, please see our results in fig. A7. These new results demonstrate that CIPHER can seamlessly integrate different infill types, including configurations with extrusion gradients. In general, we find any infill pattern that can be mathematically expressed can also be accommodated within CIPHER's framework. Notably, in certain cases, CIPHER was able to generate infills unattainable with traditional slicing software, take for example gradients introduced along the direction of the toolpath rather than perpendicular to it. We discuss these new results in our revised manuscript in pg. 14.*

<u>Comment 5.8: 8 - It would be helpful to include a figure comparing a standard part printed with the proposed method against one from a conventional setup to visually demonstrate its advantages.</u>

Authors response: *The comparison proposed in (8) has been conducted and results are shown in fig. A7. We notice that in some cases CIPHER is demonstrably better than the shape generator (generator fails for rocket, produces thin features in table) and vice versa (see duck). We discuss these observations on pg. 14.*

<u>Comment 5.9: 9 - The authors should include a basic comparison of the performance of the generation method against other relevant approaches described in the literature or add references.</u>

Authors response: *We have added a discussion noting that while generative approaches might be more generalizable, current methods lack process-awareness and we therefore use them only as fallbacks (pg. 14). This is supported by pairwise comparisons between parts obtained through primitive combinations and those produced by shape generators, in which the former mostly outperform the latter in terms of manufacturability, as shown in Extended Data Fig. A7.*

Some other minor observations:

Comment 5.10: 1 - The authors should consider selecting an alternative to the yellow color used in the figures, as it has poor contrast and is not clearly visible on a white background

Authors response: *The color schemes have been refined across the manuscript to utilize a deeper shade of yellow derived from the Okabe–Ito palette, a color set specifically designed to accommodate color vision deficiencies. This adjustment has been applied consistently to figs 1 and 2 to optimize perceptual clarity and ensure inclusive visual communication.*

Comment 5.11: 2 - For improved readability, please consider inverting the top-to-bottom order of the elements in Figure 2c.

Authors response: *We have inverted the top-to-bottom order as requested. We have also revised the colors to better match the legend on fig. 2c's left.*

Comment 5.12: 3 - The authors are advised to proofread the text within all figures for spelling errors. For instance, "lossses" in the legend of Figure 2a appears to be a typo.

Authors response: *We have reviewed our figs and their captions. We thank the reviewer for this observation.*

Comment 5.13: 4 - There seems to be an incomplete sentence or a typographical error in the main text. In Section 2.4.1, line 11, the sentence begins "For context, the command M221 controls the system's This experiment…", which needs correction.

Authors response: *Thank you for pointing this out, we have removed the incomplete sentence.*

Point-by-Point response to reviewers

Hybrid Reasoning for Perception, Explanation, and Autonomous Action in Manufacturing

Christos Margadji[1] and Sebastian William Pattinson[1]

[1]Department of Engineering, University of Cambridge, Trumpington Street, Cambridge, CB2 1PZ, United Kingdom

cm2161@cam.ac.uk, swp29@cam.ac.uk

Reviewer #1 (Remarks to the Author):

Overall the authors did a commendable job of addressing this reviewer's concerns, particularly when addressing each comment individually.

After reading the other reviews, reviewer 2 did a nice job of articulating some of my observations (reviewer 1), namely a need to emphasize the novelty. This reviewer believes that that novelty comes in the domain integration, both what is done and how it is done. The reviewers seemed to acknowledge this in the response to individual comments, however the edits to the document were less direct.

This reviewer believes additional clarifications could be made in regards to domain contextualization, but these comments are recommendations.

> ***Authors response:*** *We made additional clarifications in regards to domain contextualization: In our abstract, page 1, we have rephrased "retrieval-augmented, physics-informed reasoning for contextual grounding" into "retrieval-augmented reasoning grounded in process physics and knowledge" to clarify that the benefits of RAG come with very specific domain contextualization.*
> *We also added "We hypothesise that domain contextualisation -- implemented through the injection of problem-specific and process-relevant manufacturing and engineering knowledge -- augments CIPHER's internal process understanding and leads to more robust emerging behaviours." in the main text, page 10, followed by a clarification that the form of contextualization will depend on the problem and process being tackled.*

-Paul Witherell

Reviewer #1 (Remarks on code availability):

I've reviewed only at a high level. Previous reviews noted some hardcoded locations that have since been updated.

> ***Authors response:*** *We thank the reviewer for their valuable recommendations and feedback.*

Reviewer #2 (Remarks to the Author):

This revised manuscript presents a substantially expanded and technically more developed version of the original submission. The authors have clearly invested significant effort in addressing reviewer concerns related to experimental validation, reproducibility, and empirical scope. Several additions represent substantive improvements to the paper, particularly from a systems and engineering perspective.

Noteworthy results
The most notable improvements are:
- The release of a usable codebase and training data, which meaningfully improves transparency and supports reproducibility.
- The inclusion of a human evaluation study for control-command quality, addressing concerns about bias in LLM-based judging.
- Additional experiments demonstrating application of the framework to multiple manufacturing processes (machining, welding, vacuum forming), which strengthen claims of architectural portability.
- Clearer experimental analysis of perception accuracy, uncertainty, and catastrophic forgetting.

Taken together, these additions establish the work as a carefully engineered systems demonstration of how vision-language models can be augmented with regression-based perception and retrieval mechanisms for manufacturing tasks.

Significance and relation to prior work
The work will be of interest to researchers in intelligent manufacturing systems, applied embodied AI, and hybrid AI architectures. Its primary contribution lies in system integration and empirical demonstration, rather than in primary conceptual or algorithmic novelty.

The individual components employed (VLMs, regression heads, RAG, LoRA-based fine-tuning) are all largely well established. The novelty arises from their orchestration within a single pipeline applied to manufacturing control. This positioning is reasonable, but the manuscript would benefit from framing itself more explicitly as a systems-level contribution, rather than implying foundational advances in reasoning or physical intelligence.

> ***Authors response:*** *We have added several clarifying statements at key points in the manuscript to establish CIPHER as a systems-level contribution. The two most important additions appear in the Abstract, page 1, where we describe CIPHER as "a systems-level vision-language-action (VLA) framework," and in the Discussion, page 14, where we state that "This work introduces CIPHER, a vision-language-action (VLA) system designed to support autonomous control" and that "CIPHER represents a systems-level contribution toward […]" in page 15.*

Support for claims and conclusions
While the experimental results now convincingly support claims about practical functionality, several higher-level claims remain not yet fully substantiated.

In particular, the manuscript continues to describe the system as performing physics-informed or expert-like reasoning. However, the evidence presented does not yet clearly distinguish between:
- genuine causal or physical reasoning, and
- retrieval-driven pattern completion grounded in curated textual knowledge.

Examples such as temperature-viscosity adjustments are plausible and useful, but could also plausibly arise from retrieved heuristics rather than explicit understanding of physical relationships. The system does not currently demonstrate formal causal models, interventional reasoning, prediction of downstream effects, or temporal reasoning over process evolution.

As such, claims relating to physics-informed reasoning, industrial readiness, and expert-level understanding would benefit from further moderation relative to the evidence provided.

> ***Authors response:*** *It is indeed difficult to definitively establish the source of the system's behavior. We also agree that causal or physical reasoning and retrieval-augmented pattern completion are not equivalent. At the same time, in practice these need not be viewed as strictly separable: behavior that appears physics-informed may emerge from the model's ability to retrieve, integrate, and apply physically meaningful domain knowledge across context. In this sense, we view the system's behavior as physics-informed, even if the underlying mechanism cannot be uniquely attributed to explicit physical reasoning.*
>
> *We appreciate the reviewer's concern that some of the original phrasing could be read as overstating the present level of industrial readiness or expert-level understanding. We have therefore revised several passages to better align the claims with the evidence. For example, we have moderated the abstract statement in page 1, now referring to "potential for deployment in real industrial environments" and the introduction text in page 4 suggesting that CIPHER's articulated reasoning may support operators in high-stakes industrial decision-making, so that both now more clearly emphasize prospective utility rather than demonstrated readiness for deployment.*

Interpretation and conceptual limitations
A central concern from the original review remains only partially addressed. While the authors correctly note that manufacturing relies on domain-specific knowledge, the form of knowledge representation used here remains largely informal and unstructured:
- Manufacturing knowledge is treated as isolated text snippets retrieved via cosine similarity.
- There is no explicit semantic structure, ontology, causal graph, or systematic parameter coupling.
- The system is not grounded in established manufacturing frameworks, standards, or quality models.

This differs from how experienced manufacturing engineers typically reason in practice, who rely on formal process models, coupled parameter interactions, constraints, and standards-driven quality frameworks.

The additional experiments demonstrate broader applicability of the approach, but they do not fully resolve this underlying limitation. Generalisation here reflects portability of a systems template, not principled abstraction of manufacturing reasoning.

> ***Authors response:*** *We agree with this concern and do not claim that the current RAG-based formulation constitutes manufacturing-grade reasoning in the sense used by experienced manufacturing engineers.*
>
> *In the present work, manufacturing knowledge is represented primarily as retrieved textual context rather than as an explicit formal process model with structured*

> *semantics, coupled parameter dependencies, causal mechanisms, or standards-grounded quality abstractions. We therefore view the current approach as a portable systems template rather than a principled abstraction of manufacturing reasoning.*
>
> *To make this limitation explicit, we have now added a passage in the RAG-related discussion paragraph in page 15 clarifying that this remains an important direction for future work.*

Methodology and reproducibility
The methodology is technically sound and generally well executed. The expanded Methods section, ablation studies, and evaluation protocols meet expected standards in the field.

With the release of code, data samples, and pretrained models, the work is now largely reproducible in principle. Some elements of the full pipeline, particularly knowledge-base construction and prompt scaffolding, still rely on implicit design choices, but this is now clearly documented.

Overall assessment
The manuscript has evolved into a well-developed engineering contribution demonstrating what can be achieved with current vision-language models when augmented with domain-specific perception and retrieval in controlled manufacturing settings.

However, the conceptual limitations identified in the original review remain. To align claims with evidence, the paper would benefit from:

- Explicitly framing the system as a proof-of-concept or systems demonstration rather than a foundational advance.
- Substantially moderating claims of physics-informed or expert-like reasoning.
- Clearly discussing what would be required for manufacturing-grade reasoning, including formal knowledge representation, causal modelling, standards integration, temporal reasoning, and uncertainty quantification.

> ***Authors response:*** *these three points, namely i) representing CIPHER as a systems-level contribution, ii) moderating claims on expert-like reasoning and iii) discussing future directions for proper knowledge-injection) have been clarified following the reviewer's suggestions above.*

With these clarifications, the work would stand as a valuable and appropriately scoped contribution that delineates both the promise and current limitations of foundation models in manufacturing control.

Aydin Nassehi

Reviewer #2 (Remarks on code availability):

The codebase has been substantially improved compared to the original submission and is now organised, readable, and suitable for inspection by the research community. A clear repository structure, accompanying README, example scripts, prompt files, and geometry artefacts are provided, and obvious portability issues such as hard-coded local paths appear to have been addressed.

The released code, together with the associated data and pretrained models, supports understanding of the system design and enables partial reproduction of key components and demonstrations. While the repository is not a fully end-to-end, push-button reproduction of all experiments, it is a usable research artefact that allows independent inspection, execution of example workflows, and exploration of the proposed framework.

The code availability now meets a reasonable standard for a complex research system and represents a meaningful improvement in transparency and reproducibility relative to the initial version.

Authors response: *We thank the reviewer for their valuable feedback.*

Reviewer #3 (Remarks to the Author):

I co-reviewed this manuscript with one of the reviewers who provided the listed reports. This is part of the Nature Communications initiative to facilitate training in peer review and to provide appropriate recognition for Early Career Researchers who co-review manuscripts.

Reviewer #3 (Remarks on code availability):

The authors have made genuine improvements: refactored code, removed hardcoded paths, provided sample data and a pre-trained model on Hugging Face. The README now includes dependencies and a script table.

However, I haven't attempted installation myself. Based on inspection, the repository appears functional for researchers familiar with VLM pipelines but lacks worked end-to-end examples. The RAG knowledge base construction could be better documented.

> ***Authors response:*** *We have added a self-contained, end-to-end knowledge base construction pipeline in the knowledge_generation/ module in our repository. This is structured as a documented, multi-stage framework (hierarchy.py → facts.py → embeddings.py → visualization.py) that takes a single manufacturing topic as input (e.g. "Additive Manufacturing") and produces the complete RAG knowledge base from LLM-generated taxonomy and fact extraction, through to embedding generation and t-SNE visualisation - in a single command.*
>
> *We believe this directly addresses the concern about lacking worked end-to-end examples, as any researcher can now reproduce the full knowledge base construction process for an arbitrary manufacturing domain with a single invocation.*

In summary: adequate for academic reproducibility, though not immediately accessible to the broader community. Sufficient for motivated researchers with appropriate expertise to replicate the work.

> ***Authors response:*** *We thank the reviewer for their valuable feedback.*

Reviewer #4 (Remarks to the Author):

The authors have adequately addressed my comments.

Authors response: *We thank the reviewer for their valuable feedback.*
